# Unmet clinical needs in women with polycystic ovary syndrome in regard to mental health: a cross-sectional study

**DOI:** 10.1007/s00404-024-07452-y

**Published:** 2024-03-11

**Authors:** Marina Sourouni, Julia Estermann, Norman Bitterlich, Susanna Weidlinger, Annette Bachmann, Petra Stute

**Affiliations:** 1 Department for Gynaecological Endocrinology and Fertility Disorders, University Hospital Heidelberg, Heidelberg, Germany; 2 Faculty of Medicine, University of Bern, Bern, Switzerland; 3 Freelance Statistician, Chemnitz, Germany; 4 Department of Obstetrics and Gynaecology, University Hospital Inselspital, Friedbuehlstrasse 19, 3010 Bern, Switzerland; 5 Division of Gynaecological Endocrinology and Reproductive Medicine, Department of Gynecology and Obstetrics, University Hospital Frankfurt, Frankfurt, Germany

**Keywords:** Polycystic ovary syndrome, Unmet clinical need, Mental health, Patient satisfaction

## Abstract

**Purpose:**

Polycystic ovary syndrome (PCOS) management has hardly been standardized until recent years. Despite the existence of a detailed, evidence-based guideline published by the European Society of Human Reproduction and Embryology (ESHRE), it remains unclear to what extent healthcare providers adhere to this guideline. Our aim is to evaluate the gynaecological medical care provided in women with PCOS, particularly in terms of mental health, from the patients’ perspective.

**Methods:**

For this cross-sectional online cohort study in women with PCOS, we designed a standardized, non-validated questionnaire covering aesthetic aspects, metabolism, menstrual cycle, reproduction, mental health, and prevention of chronic non-communicable diseases.

**Results:**

Among 1879 participants, various mental health aspects were reported: body image (*n* = 1879), eating patterns/habits (*n* = 1878), and emotional well-being (*n* = 1874). Although nearly all women (99.7%) reported complaints on at least one session of mental health, consultation rates were low (body image 9.7%, eating patterns/habits 16.6%, emotional well-being 4.4%). Mean satisfaction with counselling on the different domains varied from moderate to fairly satisfying, with scores of 56.0 points (SD 31.7), 53.5 points (SD 32.0), and 63.7 points (SD 30.2), respectively. More complaints were associated with lower satisfaction. The overall satisfaction with the management provided by the healthcare practitioner (HCP) was low, averaging 36.5 points (SD 29.7). Consequently, most women wished for more counselling (58.9%).

**Conclusion:**

Women affected by PCOS are not properly managed according to ESHRE guideline in regard to mental health issues. Overall consultation rates and corresponding satisfaction with management were poor, highlighting the need for significant improvements in healthcare provision.

**Supplementary Information:**

The online version contains supplementary material available at 10.1007/s00404-024-07452-y.

## Introduction

Polycystic ovary syndrome (PCOS) is the most common endocrine disorder, afflicting females of reproductive age [1]. Its prevalence varies depending on diagnostic criteria used and is estimated between 8 and 13% with many women in the community still remaining undiagnosed [2].

Diagnostic manifestations of PCOS include oligomenorrhoea (cycle length >35 days) or amenorrhoea (absence of menstrual periods), clinical hyperandrogenism (hirsutism, acne, alopecia) and/or hyperandrogenaemia (excessive levels of free testosterone, free androgen index, or calculated bioavailable testosterone in blood), and polycystic ovary morphology (follicle number per ovary ≥20 and/or an ovarian volume ≥20 ml on either ovary). PCOS is also associated with other comorbidities including an increased risk of chronic non-communicable diseases (NCD) (e.g. cardiovascular disease, insulin intolerance, diabetes mellitus), and mental disorders (e.g. depression, anxiety, eating disorders) [3–5]. Therefore, PCOS demands a holistic, interdisciplinary management. Physicians can use the international evidence-based recommendations published from the European Society of Reproduction and Embryology (ESHRE) in 2018, as an orientation tool for the diagnosis, management, and follow-up/screenings in women with PCOS [6]. According to the ESHRE guidelines, the following screenings are recommended: weight monitoring, blood pressure, glycaemic status, cardiovascular risk, fasting lipid profile, oral glucose tolerance test (OGTT), obstructive sleep apnoea (OSA), and emotional well-being (Table 1). For emotional well-being, screening questions are suggested. Although detailed guidance is available, it remains unclear to which extent, if at all, healthcare providers (HCP) follow the ESHRE guideline. Indeed, previous studies [7–10] have reported a great dissatisfaction of women with their healthcare practitioner (HCP).

**Table 1:** Screenings recommended according to ESHRE guidelines in women with PCOS

Screening parameter	When?
Weight monitoring	Each visit, at minimum every 6–12 months
Blood pressure	Annually
Glycaemic status	Baseline, then every 1–3 years
Cardiovascular risk factors	At least once, including obesity, cigarette smoking, dyslipidaemia, hypertension, impaired glucose tolerance, lack of physical activity
Fasting lipid profile	If overweight
Oral glucose tolerance test (OGTT)	Baseline in high-risk women. For all women with PCOS, when planning a pregnancy or seeking fertility treatment, in pregnancy at 24–28 weeks gestation, if not done preconception: during pregnancy <20 weeks gestation
Obstructive sleep apnoea (OSA)	Whenever related symptoms are present (snoring, waking unrefreshed, daytime sleepiness)
Emotional well-being (screening for anxiety and depressive symptoms, psychosexual dysfunction, body image and eating disorders)	Routine screening at diagnosis

The aim of this cross-sectional cohort study was to evaluate the unmet clinical needs and patient-reported satisfaction with medical healthcare management provided to women with PCOS. We hypothesized that women with PCOS may not receive counselling in line with the ESHRE guideline, but seek for a comprehensive management in respect to hyperandrogenism, metabolism, menstrual cycle profile, reproduction, affective disorders, and NCD prevention. In this publication, we focus on the topic mental health/affective disorders. Studies have shown a high prevalence of depression, body image disturbances, and low self-esteem in women with PCOS [11, 12] and link this condition to eating disorders, such as bulimia and recurrent dieting [13].

## Materials and methods

This cross-sectional cohort study (online survey) included females aged 18 years or older, speaking German and fulfilling ESHRE PCOS diagnostic criteria [6], or having been diagnosed with PCOS by a gynaecologist. Women were excluded if they were postmenopausal, or had been diagnosed with other causes of hyperandrogenism. Recruitment occurred from 2021-01-01 to 2021-03-14. For recruitment, a flyer was created to attract the attention of affected women including the web link to the survey (supplementary file 4). The link for the online questionnaire was distributed through different online channels such as Facebook, Instagram, self-help forums, support groups, university newsletters, and hospital websites. On Facebook, it was posted on one hand on the private profile and story of the second co-author with a possibility to share, and on the other hand, in 18 different PCOS-related Facebook groups. On Instagram, it was posted on the private profile and story of the second co-author with various hashtags to distribute the content. The following Internet platforms agreed with posting the flyer on their webpage and distributing it to their members via email, Facebook, or other channels: “swissmom.ch”, “familienleben.ch”, “GLAMOUR-Forum”, “PCOS Selbsthilfe Deutschland” and “Selbsthilfe Zürich”. The following hospital groups posted the information on their intranet, newsletter, or hospital webpage: Inselspital Bern (CH), Uniklinikum (University Hospital) Münster (D), Uniklinikum Frankfurt (D). In addition, the Uniklinikum Münster (D), Frauenpraxis (Women’s Practice) Lindenhofspital Bern (CH), and Frauenpraxis Buchenhof Sursee (CH) agreed to display the flyer in their waiting room. Ultimately, the information about the study was distributed by various universities via email, newsletter, or bulletin boards to their students and members. Participating universities were the University of Bern (CH), Basel (CH), Zürich (CH), Münster (D), Frankfurt (D), ETH Zürich (CH), Fachhochschule Nordwestschweiz (CH), Zürcher Fachhochschule (CH), and pädagogische Hochschule (Pedagogical University) St. Gallen (CH). Data were collected anonymously. To avoid double entry, each participant had to set up an individual identification code using defined letters of their parents’ name, their own name, and year of birth. This is a cross-sectional online study. The Cantonal Ethics Committee Bern (Req-2020-00801) has confirmed that no ethical approval is required.

This publication focusses on the topic mental health/affective disorders. The topic hyperandrogenism and overweight/obesity from an aesthetic viewpoint has already been published [10]. The other topics will be covered in future publications.

### Questionnaire

We created an eight-domain questionnaire (Supplementary File 1, 2) through a multi-step process, including testing for comprehensibility by 15 volunteers, statistical evaluation by a statistician (NB), and a final review by the co-authors. The questionnaire covered all ESHRE guideline domains [6], including demographics, PCOS diagnostic criteria, aesthetics, metabolism, menstrual cycle, reproduction, mental health, NCD prevention, and monitoring. Mental health included body image, eating patterns, and emotional well-being. The assessment utilized the screening questions recommended in the 2018 ESHRE guideline for PCOS. These questions were based on the validated AD-EVA questionnaire, the validated PHQ questionnaire, and the validated SKOFF questionnaire [14–16].

Participants who received counselling regarding mental health rated their satisfaction on a scale of 0–100. The resulting categories were: (1) not at all satisfied (0–20 points), (2) not satisfied (21–40 points), (3) moderately satisfied (41–60 points), (4) well satisfied (61–80 points), and (5) absolutely satisfied (81–100 points). If a participant never had a consultation on a mental health aspect, she was asked if she wished for one.

Finally, participants were asked to rate their overall satisfaction with their HCPs’ management of mental health issues, express wish for additional consultations and consultation content. The other domains were addressed similarly (separate publications). To minimize information bias, questions were posed neutrally, participants were blinded to the study hypotheses, medical terminology was clarified, input validation was implemented, and the possibility to mark “unknown” was provided. The questionnaire was programmed and run in REDCap software to guarantee secure data processing.

### Statistics

**Fig. 1:**
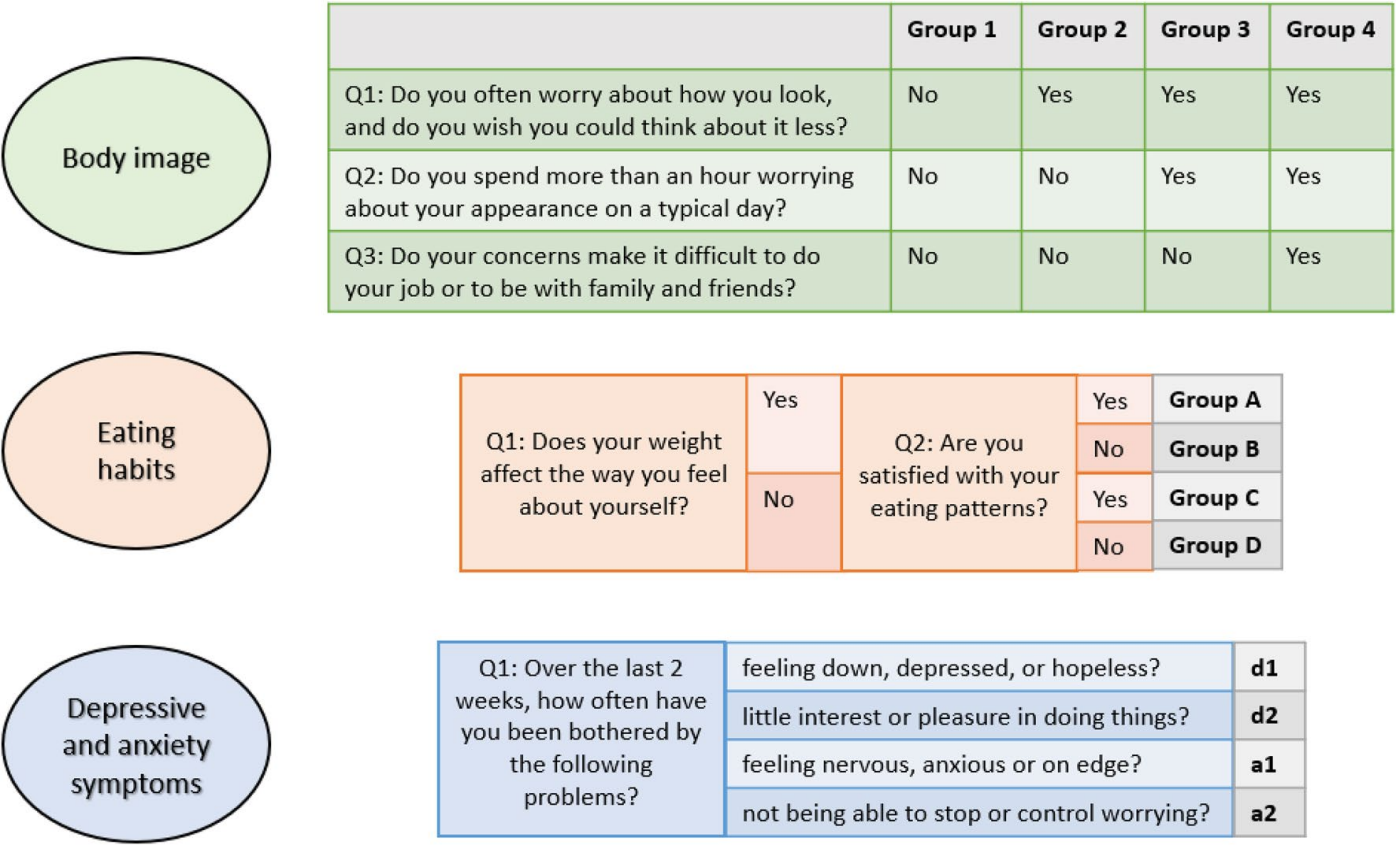
Schematic representation of the different groups for statistical analysis

Initially, 199 participants were required based on power calculations using the G*Power Software, with satisfaction measured on a scale from 0 to 100 as the parameter of interest. The goal was to detect a minimum difference of 10% in observed frequencies from an expected frequency of 50%. Since expected frequencies can vary, we selected the value of 50% in the power calculation, necessitating the highest number of cases. However, data collection was not halted after reaching 199, but continued for a total of 10 weeks. Descriptive statistics were used to describe the study cohort. For each domain of mental health assessed, a subgroup analysis followed. The original questionnaire in German language is provided in Supplementary File 2. To facilitate interpretation of the data discussed in this publication, the relevant aspects will be presented in English. Three questions were used to assess worries about body image: Do you often worry about how you look and do you wish you could think about it less? Do you spend more than an hour worrying about your appearance on a typical day? Do your concerns make it difficult to do your job or to be with family and friends? The third question followed only if the second question was answered in the affirmative. While assessing body image, participants were further presented with an illustration and asked to evaluate their self-perceived image and desired body image using a numerical scale from 1 (lean figure) to 6 (non-lean figure). For the data analysis regarding body image, the cohort was divided into four groups: those with no concerns (group 1), those with general concerns (first question answered with yes, group 2), those with extensive appearance-related worries (first two questions answered with yes, group 3), and those who answered all three questions with yes (group 4) (Fig. 1).

To assess eating patterns/habits, women were asked if their weight plays an important role in the way they feel and if they are satisfied with their eating habits. Depending on the answers, the cohort was again divided into four groups. Women for whom weight plays an important role (first question answered with yes) were divided into group A and B depending on their satisfaction with their eating habits (satisfied group A, not satisfied group B). Analogously, women for whom the weight did not play an important role (first question answered with no) were divided into group C and D (satisfied group C, not satisfied group D) (Fig. 1).

In a next step, women were asked how often in the last 2 weeks they feel down, depressed, or hopeless (d1), have little interest or pleasure in doing things (d2), feel nervous, anxious, or on edge (a1), or are not able to stop or control worrying (a2) (Fig. 1). Answers varied in a scale of 1 = never, 2 = rarely, 3 = sometimes, 4 = often, and 5 = constantly. A combined mean frequency of all bothersome feelings was used to describe the extent of affective disorder. This was calculated using the sum of frequencies of the four individual symptoms divided by four [(d1 + d2 + a1 + a2)/4]. The resulting groups depending on the total score had bothersome feelings: (1) never (1.0–1.5), (2) rarely (>1.5–2.5), (3) sometimes (>2.5–3.5), (4) often (>3.5–4.5), and (5) constantly (>4.5–5.0).

Descriptive statistics and statistical tests to compare groups including Mann–Whitney *U* test, Chi-square test, Jonckheere–Terpstra test, and Pearson correlation coefficient (*r*) were applied to analyse the data as necessary. A level of *p* < 0.05 was considered significant. Data were analysed using SPSS software version 27.0.

## Results

### Characteristics of the cohort

**Fig. 2:**
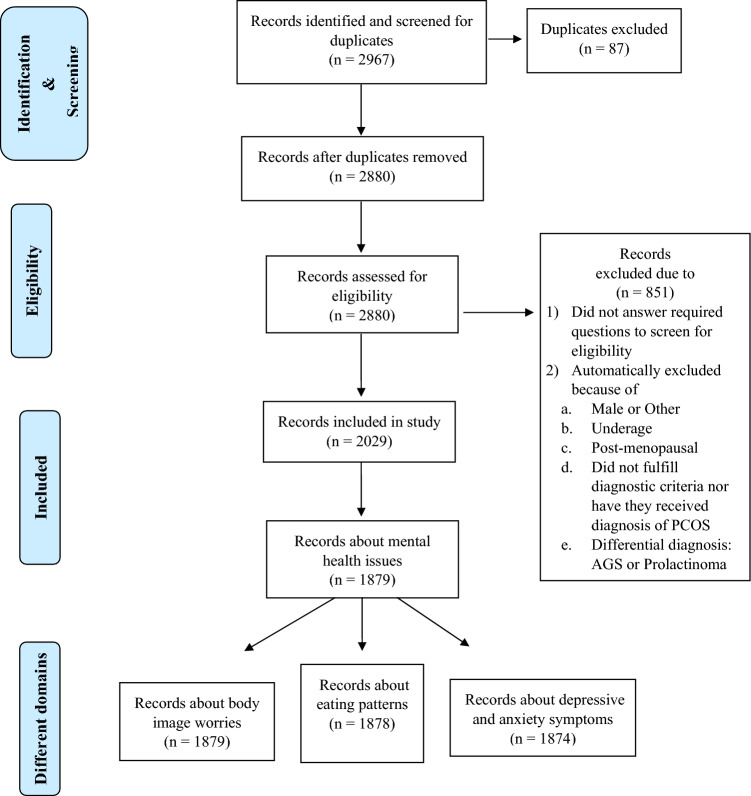
Flowchart of the record screening process

Overall, 2967 data entries were registered. After checking for double entry and eligibility criteria, a total of 2029 participants were included in the final cohort (Fig. 2). 1720 women (84.8%) reported a PCOS diagnosis by their HCP. The remainder fulfilled PCOS diagnostic criteria without former diagnosis. For this publication, 1879 individuals (92.6%) provided data on at least one mental health domain (Table 2). Most women lived in Germany (72.4%), were childless (70.6%), well educated (61.6%), and worked at least part-time (68.3%). Mean age was 28.9 ± 5.8 years. Mean BMI was 30.49 ± 8.5 kg/m^2^ with 48.6% of the participants being obese.

**Table 2:** Characteristics of the cohort (*n* = 1879)

Age (years)	Mean (SD)	28.9 (5.8)
Domicile, *n* (%)	Switzerland	374 (20.0)
	Germany	1361 (72.4)
	Austria	121 (6.4)
	Other	23 (1.2)
Marital status, *n* (%)	Married or registered partnership	749 (39.9)
	Divorced	43 (2.3)
	Widowed	1 (0.1)
	Single	1040 (55.3)
	No answer	46 (2.4)
Children number, *n* (%)	None	1326 (70.6)
	1	323 (17.2)
	2	178 (9.5)
	>2	52 (2.8)
Highest level of education completed, *n* (%)	No degree	0 (0)
	Secondary level 1 (=obligatory)	437 (23.3)
	Secondary level 2, universal education	320 (17.0)
	Secondary level 2, vocational education	283 (15.1)
	Tertiary level, high vocational education	174 (9.3)
	Tertiary level, university	664 (35.3)
	Other	1 (<0.1)
Current employment status, *n* (%), multiple answers possible	Full-time	802 (42.7)
	Part-time	505 (26.9)
	Hourly wage	128 (6.8)
	Self-employed	68 (3.6)
	Student	474 (25.2)
	Not employed	122 (6.5)
Ethnicity, *n* (%)	White (only or in combination)	1746 (92.9)
	Other: Hispanic, Arabic, Asian, Black	133 (7.1)
Height (cm)	Mean (SD)	167.4 (6.4)
Weight (kg)	Mean (SD)	85.6 (25.0)
BMI (kg/m^2^)	Mean (SD)	30.5 (8.5)
Weight class, *n* (%)	Underweight (BMI < 18.5)	39 (2.1)
	Normal weight (BMI 18.5 to <25.0)	582 (31.0)
	Overweight (BMI 25.0 to <30.0)	342 (18.2)
	Obesity Class I (BMI 30.0 to <35.0)	363 (19.3)
	Obesity Class II (BMI 35.0 to <40.0)	273 (14.5)
	Obesity Class III (BMI ≥ 40)	279 (14.8)

*SD* standard deviation, *BMI* body mass index

### Aspect ‘worries about body image’

The questionnaire included three questions to capture the attitude of the women in regard to their body image. Of the 1879 women, 80% admitted to worry a lot about the way they look and wished to be able to think less about it (general concern, question 1) and 46.4% confessed spending more than 1 h per day worrying about their appearance (question 2). Such worries made it hard for 41.5% of these women (*n* = 1521) to do their job or be with family and friends (question 3). The majority of women defined their self-perceived image with a score of ≥4 (59.9%) and their desired body image as 2 or 3 (91.1%) (see appendix/supplementary data, file 2, section 5—mental health and emotional well-being, record id 322). Almost 82% of the women wished for a leaner figure (greater number of ‘is’—than ‘wished’—figure) and only 16.2% of the women were satisfied with their figure (number ‘is’ = number ‘wished’figure).

The total cohort was divided into four groups for the subgroup analysis (see ‘Statistics’, Fig. 1). Thereby it seems that as concerns regarding body image rise stepwise (from group 1 to group 4), so do the BMI, the discrepancy between self-perceived and desired body image, as well as the wish for consultation if no consultation was offered (Table 3, *p* < 0.001). The satisfaction of the 183 women counselled about body image was moderate (56.0 ± 31.7). Thereby, women with no concerns were the most satisfied (64.0 ± 33.5), while women with great concerns were the least satisfied (44.7 ± 30.2, *p* = 0.025) (Table 3). Interestingly women with more concerns were not significantly more often counselled about body image (*p* = 0.220), but wished significantly more often (74%) for a consultation than women with no concerns (14%, *p* < 0.001).

**Table 3:** Subgroup analysis regarding worries about body image

Group* (*n*, % of total cohort)	Group 1 (358, 19.0)	Group 2 (522, 27.8)	Group 3 (499, 26.6)	Group 4 (500, 26.6)	*p* value
Mean age (SD)	29.2 (5.8)	28.6 (5.2)	29.0 (5.5)	29.0 (5.5)	0.831^#^
Mean BMI (SD)	25.3 (6.6)	29.4 (7.5)	31.8 (8.4)	34.1 (8.7)	<0.001^a^
Satisfied with body image (*n*, % of group)	173 (48.3)	65 (12.5)	38 (7.6)	28 (5.6)	<0.001^b^ 0.206^c^
Wished leaner figure (*n*, % of group)	171 (47.8)	446 (85.4)	458 (91.8)	468 (93.6)	<0.001^b^ 0.277^c^
Received consultation (*n*, % of group)	29 (8.1)	47 (9.0)	54 (10.8)	53 (10.6)	0.241^b^
Mean satisfaction with consultation (*n*, % of group)	64.0 (33.5)	58.8 (33.7)	57.3 (29.3)	47.7 (30.2)	0.025^b^
No prior consultation, but wish for consultation (*n*, % of group)	50/328 (15.2)	204/475 (42.9)	246/445 (55.3)	331/447 (74.0)	<0.001^a^

^*^Group 1: all questions answered with no. Group 2: first question answered with yes. Group 3: first two questions answered with yes. Group 4: all three questions answered with yes

^#^Kruskal–Wallis test

^a^Value refers to all pairwise comparisons (group 1 vs. 2, 1 vs. 3, 2 vs. 3, 1 vs. 4)

^b^Value refers to comparison of group 1 with group 4 (no concern vs great concern)

^c^Value refers to comparison of group 3 with group 4

### Aspect ‘eating patterns/habits’

In the next section about eating patterns/habits, women were asked if weight affects the way they feel about themselves (question 1) and if they were satisfied with their eating patterns (question 2). All women who responded to the section body image reported also on the session eating patterns except one (*n* = 1878) (Fig. 2).

A substantial portion, 88.3% (1659 women), acknowledged that their weight impacts their self-perception (question 1), and 62.6% (1175 women) admitted dissatisfaction with their eating habits (question 2). Only 311 women (16.6%) were counselled on eating patterns/habits with a moderate satisfaction (53.5 ± 32.0). The total cohort was divided into four groups for the subgroup analysis (see ‘Statistics’, Fig. 1). The great majority of women (*n* = 1114, 59.3%) were not satisfied with their eating habits, and weight played an important role in the way they feel (group B). The results referring to women whose emotional well-being depends on body weight (group A and B) are depicted in Table 4. Interestingly, women dissatisfied with their eating habits were not more likely to receive body image counselling, they were less satisfied with the provided consultation (*p* = 0.020), and expressed a significantly stronger desire for counselling compared to those with no concerns (66.1 vs. 45.3%, *p* < 0.001, Table 3). Women whose emotional well-being was not influenced by body weight were the ones with the highest (when satisfied with their eating habits, group C) or the lowest (when not satisfied with eating habits, group D) mean satisfaction with a score of 72.0 ± 27.0 and 44.1 ± 29.2, respectively (*p* = 0.040).

**Table 4:** Subgroup analysis regarding eating patterns/habits

Group (*n*, % of total cohort)	Group A (545, 29%)	Group B (1114, 59.3%)	*p* value
Mean age (SD)	28.6 (5.7)	29.3 (5.4)	0.002
Mean BMI (SD)	28.4 (8.0)	32.6 (8.2) *n* = 1113	<0.001
Received consultation (*n*, % of group)	112 (20.6)	181 (16.2)	0.220
Mean satisfaction with consultation (*n*, % of group)	58.6 (32.2)	49.5 (31.7)	0.020
No prior consultation, but wish for consultation (*n*, % of group)	196/433 (45.3)	617/933 (66.1)	<0.001

Group A: emotional well-being depends on body weight, satisfied with eating habits

Group B: emotional well-being depends on body weight, not satisfied with eating habits

In total, 875 women (46.6%) would have wished for a consultation, with the majority (*n* = 617, 70.5%) falling in group B (weight affects well-being, not satisfied with eating habits).

Women of group B had also the highest mean BMI (32.6 ± 8.2), while women of group C had the lowest mean BMI (22.9 ± 5.4) (*p* < 0.001).

### Aspect ‘depressive and anxiety symptoms’

In total, 1874 women reported on frequency of depressive (d1, d2) and anxiety (a1, a2) symptoms. Additionally, based on their answers, a mean frequency of all bothersome feelings was calculated to describe the extent of affective disorder (see ‘Statistics’).

The number of women reporting any frequency of the different bothersome feelings in relation to body image concerns is presented in Table 5.

**Table 5:** Distribution of women reporting any frequency of the different bothersome feelings (d1, d2, a1, a2) in correlation to the extent of worries about body image (group 1, 2, 3, 4)

Frequency	1 = never	2 = rarely	3 = sometimes	4 = often	5 = constantly	Total (*n*)
Over the last two weeks, how often did you feel						
*A) down, depressed or hopeless (d1)*						
Group1 (*n*, % of group)	117 (32.8)	153 (42.9)	55 (15.4)	19 (5.3)	13 (3.6)	357
Group2 (*n*, % of group)	82 (15.8)	218 (41.9)	108 (20.8)	69 (13.3)	43 (8.3)	520
Group 3 (*n*, % of group)	34 (6.8)	158 (31.7)	146 (29.3)	94 (18.8)	67 (13.4)	499
Group 4 (*n*, % of group)	4 (0.8)	73 (14.7)	131 (26.3)	142 (28.5)	148 (29.7)	498
Total (*n*, %)	237 (12.6)	602 (32.1)	440 (23.5)	324 (17.3)	271 (14.5)	1874
*B) having little interest or pleasure in doing things (d2)*						
Group 1 (*n*, % of group)	93 (26.1)	173 (48.5)	52 (14.6)	30 (8.4)	9 (2.5)	357
Group 2 (*n*, % of group)	102 (19.6)	219 (42.1)	98 (18.8)	70 (13.5)	31 (6.0)	520
Group 3 (*n*, % of group)	46 (9.2)	160(32.1)	143 (28.7)	103 (20.6)	47 (9.4)	499
Group 4 (*n*, % of group)	10 (2.0)	68 (13.7)	136 (27.3)	153 (30.7)	131 (26.3)	498
Total (*n*, %)	251 (13.4)	620 (33.1)	429 (22.9)	356 (19.0)	218 (11.6)	1874
*C) nervous, anxious or on edge (a1)*						
Group 1 (*n*, % of group)	94 (26.3)	124 (34.7)	72 (20.2)	43 (12.0)	24 (6.7)	357
Group 2 (*n*, % of group)	94 (18.1)	156 (30.0)	119 (22.9)	92 (17.7)	59 (11.3)	520
Group 3 (*n*, % of group)	34 (6.8)	159 (31.9)	106 (21.2)	123 (24.6)	77 (15.4)	499
Group 4 (*n*, % of group)	11 (2.2)	61 (12.2)	110 (22.1)	152 (30.5)	164 (32.9)	498
Total (*n*, %)	233 (12.4)	500 (26.7)	407 (21.7)	410 (21.9)	324 (17.3)	1874
*D) not being able to stop or control worrying (a2)*						
Group 1 (*n*, % of group)	159 (44.5)	97 (27.2)	49 (13.7)	31 (8.7)	21 (5.9)	357
Group 2 (*n*, % of group)	144 (27.7)	168 (32.3)	99 (19.0)	61 (11.7)	48 (9.2)	520
Group 3 (*n*, % of group)	71 (14.2)	152 (30.5)	108 (21.6)	96 (19.2)	72 (14.4)	499
Group 4 (*n*, % of group)	20 (4.0)	58 (11.6)	114 (22.9)	132 (26.5)	174 (34.9)	498
Total (*n*, %)	394 (21.0)	475 (25.3)	370 (19.7)	320 (17.1)	315 (16.8)	1874

The mean frequency of all bothersome symptoms separately as well as the total mean frequency rose with increasing concerns about body image and was the highest for women expressing great concerns (group 4). This trend was overall statistically significant (*p* < 0.001, Table 6).

**Table 6:** Mean frequency of bothersome symptoms in correlation to the extent of worries about body image

Mean frequency of:	Group 1 (*n* = 357)	Group 2 (*n* = 520)	Group 3 (*n* = 499)	Group 4 (*n* = 498)	*p* value
d1 (SD)	2.0 (1.0)	2.6 (1.2)	3.0 (1.1)	3.7 (1.1)	<0.001
d2 (SD)	2.1 (1.0)	2.4 (1.1)	2.9 (1.1)	3.7 (1.1)	<0.001
a1 (SD)	2.4 (1.2)	2.7 (1.3)	3.1 (1.2)	3.8 (1.1)	<0.001
a2 (SD)	2.1 (1.2)	2.4 (1.3)	2.9 (1.3)	3.8 (1.2)	<0.001
all feelings (SD)	2.1 (0.9)	2.5 (1.0)	3.0 (1.0)	3.7 (1.0)	<0.001

The mean frequency of all depressive and anxiety feelings rose with greater BMI, and women with obesity class II (BMI ≥ 35) (d1, d2, a2) or class III (BMI ≥ 40) (a1) reported significantly more often such symptoms than non-obese women (*p* < 0.001). Women dissatisfied with their eating habits (group B) were also significantly more often confronted with depressive (d1, d2) and anxiety (a1, a2) symptoms than women who were satisfied with their eating patterns (group A) (*p* < 0.001).

The number of women with any total mean frequency of depressive and anxiety feelings depending on worries about body image is depicted in Table 7. The great majority of the total cohort (1335, 71.2%) reported a total score of up to 3.5 (sometimes), while in the subgroup of women with great concerns about body image the great majority (287, 57.6%) reported a total score of more than 3.5 (at least often).

**Table 7:** Distribution of women with any total mean frequency of depressive and anxiety feelings in correlation to the extent of worries about body image

Frequency:	Never	Rarely	Sometimes	Often	Constantly	Total
Group 1 (*n*, % of group)	122 (34.2)	140 (39.2)	63 (17.6)	29 (8.1)	3 (0.8)	357
Group 2 (*n*, % of group)	105 (20.2)	214 (41.2)	112 (21.5)	73 (14.0)	16 (3.1)	520
Group 3 (*n*, % of group)	33 (6.6)	163 (32.7)	172 (34.5)	106 (21.2)	25 (5.0)	499
Group 4 (*n*, % of group)	1 (0.2)	70 (14.1)	140 (28.1)	185 (37.1)	102 (20.5)	498
Total (*n*, %)	261 (13.9)	587 (31.3)	487 (26.0)	393 (21.0)	146 (7.8)	1874

Resulting categories of mean frequency of all bothersome symptoms: Never (1.0–1.5), rarely (>1.5–2.5), sometimes (>2.5–3.5), often (>3.5–4.5), 5) constantly (>4.5–5.0)

Only 83 women (4,4%) were counselled on depressive and anxiety feelings with a mean satisfaction of 63.7 ± 30.2 (well satisfied). Among them, women who never had such feelings were the most and women who constantly have such feelings the least satisfied, but the difference was not statistically significant (74.4 ± 30.9 vs 59.5 ± 35.8, *p* = 0.270). A total of 930 women (52.0%) would have wished for a consultation, the majority of them (*n* = 665, 71.5%) having such symptoms at least sometimes.

### Overall satisfaction with the medical care regarding mental health

Overall, most participants wished additional consultation (*n* = 1099, 58.6%), especially more consultation time and reassurance (89.4%), more treatment options (69.2%), more sources of information, e.g. booklets (51.6%), and more examination, e.g. laboratory testing and imaging (47.0%). More than a quarter (25.8%, *n* = 290) wished all of the above.

The percentage of women wishing additional consultation gradually increased with more worries about body image and was statistically significantly higher in women having any concerns about body image (group 2, 3, 4) in comparison to women with no such concerns (group 1) (49.2, 61.9, 78.7 vs. 39.8%, *p* < 0.001). Also, women for whom body weight played an important role in emotional well-being, but who were not satisfied with their eating habits (group B), were more likely to wish for additional consultation in comparison to women who were satisfied with their eating patterns (group A) (61.7 vs. 56.3%, *p* = 0.032).

Women who reported depressive or anxiety symptoms at least sometimes (>2.5) wished significantly more often for additional consultation in comparison to the other women (73.2 vs. 41.0%, *p* < 0.001). This tendency of increasing wish for additional consultation with increasing total mean frequency of anxiety and depressive symptoms was evident in each step (sometimes vs often: 65.7 vs. 78.4%, *p* < 0.001, often vs constantly: 78.4 vs. 84.2%. *p* = 0.130).

**Fig. 3:**
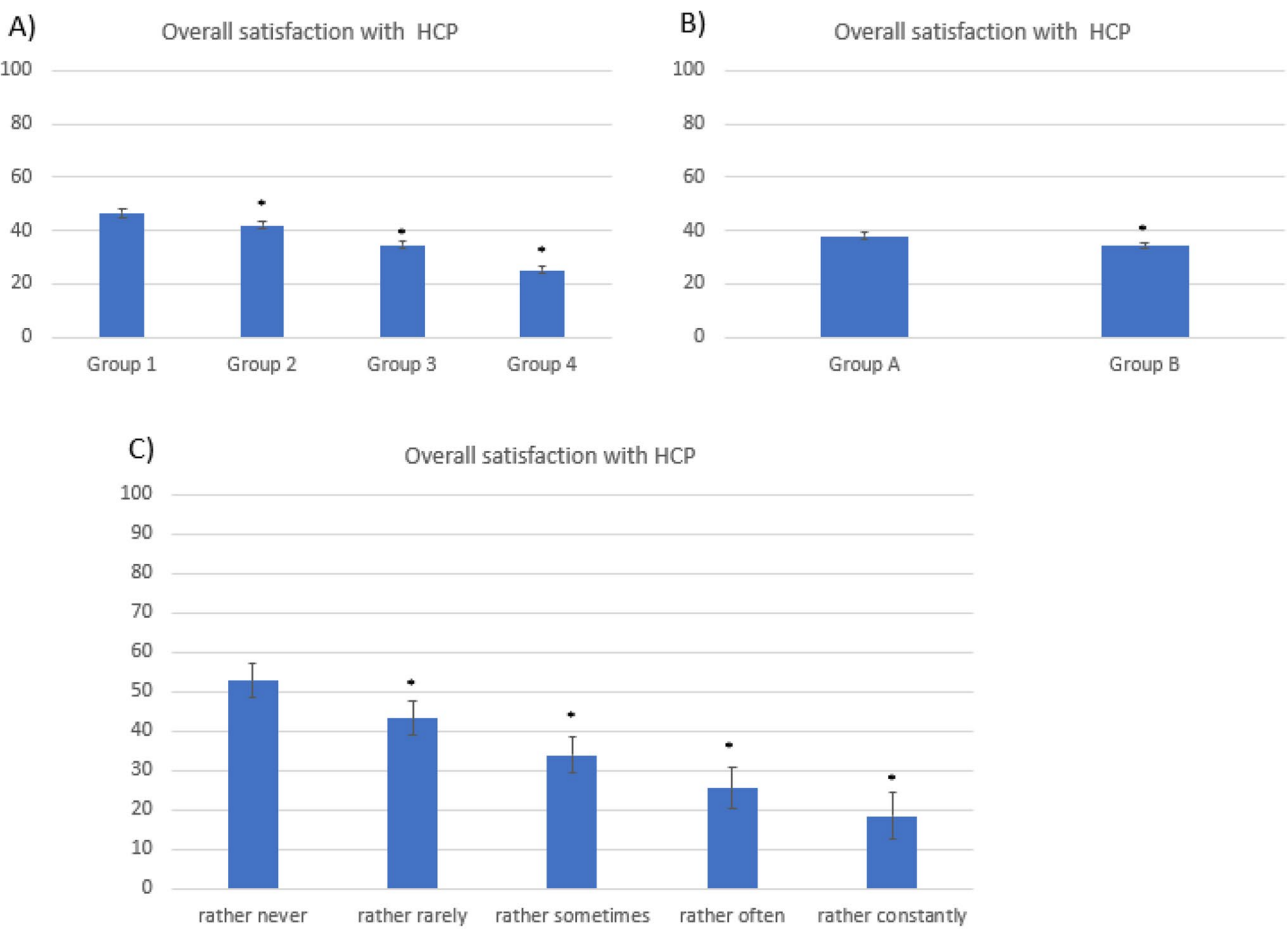
Overall satisfaction (mean ± SEM) with gynaecologist regarding mental health issues (scale 0–100) depending on **a** concerns about body image, **b** satisfaction with eating patterns, and **c** mean frequency of depressive and anxiety symptoms. *Denotes statistical significance in comparison to all other groups (*p* < 0.05)

Overall satisfaction with HCP management of complaints related to mental health was low (36.5 ± 29.7 points). More worries about body image, greater frequency of anxiety and depressive symptoms, as well as lack of satisfaction with eating habits were significantly correlated with a lower level of satisfaction (Fig. 3).

Total satisfaction did not correlate with age (*r* = −0.023, *p* = 0.310) and correlated only slightly with BMI (*r* = −0.109, *p* < 0.001), with non-obese women being significantly more satisfied than women with obesity class II or III (36.9 ± 30.3, vs 34.0 ± 29.0 vs 30.8 ± 28.8, *p* < 0.05). Overall, satisfaction was the highest in Switzerland (48.2 ± 28.9) and Austria (43.9 ± 31.7) and in these two counties significantly greater than in Germany (32.6 ± 28.8, *p* < 0.001).

## Discussion and conclusions

This online survey indicates that women with PCOS are insufficiently screened and counselled for mental health issues, despite clear recommendations from ESHRE and the Society of Polycystic Ovary Syndrome [6, 17]. Consultation rates were low, and satisfaction was moderate.

More specifically, health professionals and women should be aware that features of PCOS can impact on body image. The physicians should identify and address any concern of the patient. Furthermore, they should be aware of the increased prevalence of eating disorders and disordered eating associated with PCOS [6]. Nevertheless, only 9.7 and 16.6% of the women were counselled about body image and eating habits, respectively.

Additionally, women with PCOS should be screened for anxiety and depressive symptoms at diagnosis [6, 17]. Our survey supports this recommendation as 53.6% (a2, d2)−60.9% (a1) reported depressive and anxiety symptoms at least sometimes and 11.6% (d2)−17.3% (a1) even constantly. However, the consultation rate was very low at 4.4%.

Women expressing any concerns about body image reported significantly more often depressive and anxiety symptoms (Table 5), suggesting a co-occurrence of mental health aspects. The more the number of affected women within a mental health session, the less satisfied they were with counselling offered and the stronger the wish for counselling if none was offered. A vulnerable group demanding special focus are women reporting complaints on several aspects of mental health simultaneously as well as women with obesity class II or III. These women are the most dissatisfied with the given clinical practice and who mainly wish more consultation and care from their physician. It has to be mentioned that a greater body dissatisfaction in comparison to healthy control was observed even after adjusting for BMI [18], while symptoms of anxiety and depression in obese women with PCOS are more related to obesity than the PCOS status [19].

Studies show a high prevalence of depression, body image disturbances, and low self-esteem in women with PCOS [11, 12]. Therefore, it has been suggested that the prognosis for patients would improve by liaison between gynaecologist and psychiatrist [11]. Our survey showed also evidence of the great extent of mental health complaints in women with PCOS and aligns with the findings of these studies. Indeed, almost all women (99.7%, *n* = 1874) reported complaints in at least one symptom category of mental health and the great majority in all three sessions (77.5%, *n* = 1457). Not surprisingly, as counselling was rare, overall satisfaction with its management was very low (36.5 ± 29.7 points). The highest overall satisfaction—still only moderate—was reported by women who had less complaints. Thus, consultation efforts should primarily focus on women with more complaints.

Need for improvement of PCOS management has been described in the literature. Lately, data analysis of the same cross-sectional study regarding the aesthetic manifestations of PCOS (acne, alopecia, hirsutism, overweight/obesity) revealed low consultation and satisfaction rates with PCOS management [10]. Most women wished for more counselling regarding the aesthetic aspects of PCOS (80.8%), as well as more diagnostic (63.2%) and therapeutic options (70.2%) [10]. A recent Canadian study focussing on the experience of women with PCOS highlighted fields of improvement comparable to those arising from our survey. These included deeper knowledge and greater PCOS awareness among HCP as well as wish for more reassurance, PCOS specialists, and information tools [20]. The little priority given to medical education on reproductive endocrinology among gynaecologists could partially explain the insufficient overall management of PCOS, reflected in our survey. Gynaecologists could benefit from an improved understanding of PCOS during their residency training [7].

To include many women with PCOS in German-speaking countries, the survey was spread through social media and online platforms. Although data from women of various backgrounds were obtained, potential bias in the participants reached cannot be excluded. On the other hand, despite distributing the link via universities, women with tertiary education were not overrepresented. As the survey focussed on women’s subjective perspective, it lacks the perspective of HCP and all results and interpretations assumed truthful answers. As most participants were Caucasians, other ethnicities were underrepresented in our cohort. The questions assessing the different aspects of mental health (body image, well-being, depression) were derived from established validated questionnaires (AD-EVA questionnaire, PHQ questionnaire, SKOFF questionnaire) to provide credibility and reliability. However, it is important to note that these questions were modified as per the 2018 ESHRE guideline for PCOS, with only specific sections utilized rather than the entire questionnaire. This adaptation may impact the consistency and precision of the collected data and could result in a potential lack of insight into physiological intricacies.

Moreover, while our study provides valuable insights into the relationship between PCOS and body image, it is important to acknowledge a limitation in our assessment approach. In our investigation, we focussed solely on the external dimension of body image, specifically examining body appearance and shape. However, body image is a multifaceted construct encompassing not only physical attributes, but also functionality, internalized beauty standards, and other psychosocial factors [21, 22]. Moreover, it can be affected by chronic illness and pain, for example in individuals with cancer [23–25]. By limiting our analysis to the visual aspect of body image, we may not have captured the full complexity of this construct.

Another limitation of our study pertains to the scope of inquiry into mental health issues among participants. The ESHRE guideline recommends that gynaecologists screen patients with PCOS for mental health concerns. In our survey, we addressed this recommendation by asking participants about the presence of mental health symptoms and whether they received counselling from their gynaecologists. However, it is important to note that we did not specifically enquire whether participants proactively raised mental health issues during their medical care. This omission may impact the comprehensive assessment of mental health discussions within the healthcare setting.

The strength of our study lies in its large sample size, which allows us to assume that our results are representative. Furthermore, most studies only focus on infertility, thereby ignoring other PCOS aspects [26–28]. In this study, data were collected on all main areas (separate publications).

In conclusion, women with PCOS are seeking recognition and awareness, as evidenced by the significant participation in this online survey. The overall findings highlight the insufficiency of healthcare services in addressing the mental health aspects of women with PCOS. The consultation rate was low and satisfaction with provided management only moderate. It is imperative for healthcare providers to be vigilant in screening for mental health concerns and to provide appropriate counselling. Education about the disease and its mental health implications should be given equal emphasis as infertility counselling, A holistic PCOS management approach is not only warranted, but also highly desirable to ensure the well-being and overall health of women with PCOS.

### Supplementary Information

Below is the link to the electronic supplementary material.

Supplementary file1 (PDF 931 KB)

Supplementary file2 (PDF 381 KB)

Supplementary file3 (PDF 703 KB)

Supplementary file4 (PDF 626 KB)

Supplementary file5 (PDF 1453 KB)

## Data Availability

The data is currently not available as we still do analyses.
