# Supplementary material for: Unmet clinical needs in women with polycystic ovary syndrome in regard to mental health: a cross-sectional study
Source: Arch Gynecol Obstet. 2024 Mar 11;309(5):2115–26. doi: 10.1007/s00404-024-07452-y (PMC11018694; doi:10.1007/s00404-024-07452-y)
Supplement: Supplementary file 1 — Supplementary file1 (PDF 931 KB) [file 404_2024_7452_MOESM1_ESM.pdf]

Archives of Women's Mental Health

Unmet clinical needs in women with polycystic ovary syndrome in regard to mental health – a cross-sectional study

Sourouni Marina, M. D, Estermann Julia, M. D., Bitterlich Norman, Ph. D, Weidlinger Susanna, M. D., Bachmann Annette, M. D., Stute Petra, M.D.

Corresponding author:

Professor Dr. med. Petra Stute, M. D.

Department of Obstetrics and Gynaecology

University Hospital Inselspital

Friedbuehlstrasse 19, 3010 Bern, Switzerland

E-mail: petra.stute@insel.ch

Telephone: (00)41-31-632-1303

Fax: (00)41-31-632-1305

ORCID:0000-0002-5591-1552

File S1: Codebook

REDCap provided by CTU Bern, University of Bern

L1375_Versorgung_PCOS

Data Dictionary Codebook

29-12-2020 14:50

#	Variable / Field Name	Field Label<br>*Field Note*	Field Attributes (Field Type, Validation, Choices, Calculations, etc.)
Instrument: **consent** (einverstndnis) Enabled as survey			
1	record_id	Record ID	text
2	info0	Welcome to this survey! Please read all information provided below carefully and give your consent to start the questionnaire.	descriptive
3	info1	**What is PCOS?** Polycystic ovary syndrome (PCOS) is the most common endocrine disorder in women of reproductive age. Typical symptoms include irregular to no menstrual bleedings and signs of hyperandrogenism such as hair loss, excess hair growth and acne.<br>**Why this study?** The aim of this study is to assess implementation of new guidelines for management of PCOS by gynecologists. We are also interested in the areas in which women with PCOS would like additional care. To achieve this we need your honest opinion.<br>**Participation Criteria**: All German-speaking women with PCOS who are of full age and have not yet reached menopause can participate in this study. If you have not yet been diagnosed with PCOS, but suspect from your symptoms that you may be affected, you are also welcome to participate. If you do not meet inclusion criteria, this will be indicated in the first part of the survey and the survey will be closed.<br>**What do you get out of it?** You make an important contribution to improving the care situation for women with PCOS. If you are interested in the results of this study, you will be informed how to subscribe for it at the end of the survey.<br>**Ethical approval and anonymity**: This study has been deemed safe by the relevant ethical committees. There are questions about your health, but no personal, identifiable data will be collected. The data are therefore collected anonymously. This means that your answers can not be traced back to you. Thus, in the first section you are instructed to create a personal code. This ensures that each person participates in the survey only once.<br>**Important information**: The survey takes about 15-20 minutes to complete. You can canel participation at any time and without giving reasons. We would like to point out that we can only us fully completed surveys for the study. Please read the questions carefully and answer them as correctly as possible. Below the answer field you will find helpful extra information or instructions in some cases.	descriptive
4	info2	If you agree please select „yes“ and the survey can begin. Thank you very much for your participation!	descriptive
5	agree	Do you give your consent to take part in this study?	radio, Required<br>1 Yes<br>0 No
6	info_submit	Please always select "Submit", to get to the next page.	descriptive
7	einverstndnis_complete	Section Header: *Form Status*<br>Complete?	dropdown<br>0 Incomplete<br>1 Unverified<br>2 Complete

Instrument: **characteristics** (charakteristika) Enabled as survey

8	participant_id	Section Header: *Personal-ID*<br>Section Header: Teilnehmer-ID<br>Please create your personal identification code in CAPITAL LETTERS out of following information:<br>-first and last letter of your mother's first name<br>-first and last letter of your father's first name<br>-first and last letter of your first name<br>-last two digits of your birth year<br>You can find an example below the answer field. If you can not give one of the details (e.g. you do not know the name of a parent) write «XX» in the correspondent position.<br>*E.g.: AETNMA76 (mother Anne, father Thorsten, you Michaela, born 1976)*	text, Required
9	info_continuation	To continue later: You can pause the survey at any time and resume it later. To do so, select „Safe & Return Later". You will then receive a code, which you can use to continue the survey at the same point. Your answers will be saved.	descriptive
10	sex	Section Header: *characteristics*<br>sex	radio, Required<br>1 female<br>2 male<br>88 other
11	sex_88<br>Show the field ONLY if: [sex] = '88'	sex - other<br>*Please specify.*	notes, Required
12	excluded_1<br>Show the field ONLY if: [sex] = '2' or [sex] = '88'	Unfortunately, you do not fulfill required criteria for this study. Nevertheless, we thank you for your willingness and wish you all the best. Please select «submit». Afterwards you can close the window.	descriptive
13	age<br>Show the field ONLY if: [sex] = '1'	age	text (integer, Min: 1, Max: 110), Required
14	excluded_2<br>Show the field ONLY if:<br>[age] = 1 or [age] = 2 or [age] = 3 or [age] = 4 or [age] = 5 or [age] = 6 or [age] = 7 or [age] = 8 or [a ge] = 9 or [age] = 10 or [age] = 1 1 or [age] = 12 or [age] = 13 or [age] = 14 or [age] = 15 or [age] = 16 or [age] = 17	Unfortunately, you do not fulfill required criteria for this study. Nevertheless, we thank you for your willingness and wish you all the best. Please select «submit». Afterwards you can close the window.	descriptive
15	height<br>Show the field ONLY if: [sex] = '1' and [age] >= 18	height [cm]<br>*e.g.: 165*	text (integer, Min: 50, Max: 250), Required
16	weight<br>Show the field ONLY if: [sex] = '1' and [age] >= 18	weight [kg]<br>e.g.*: 80*	text (integer, Min: 30, Max: 300), Required
17	bmi<br>Show the field ONLY if: [sex] = '1' and [age] >= 18	BMI<br>*Calculated automatically.*	calc, Required<br>Calculation:<br>[weight]*10000/([height]
18	domicile<br>Show the field ONLY if: [sex] = '1' and [age] >= 18	residence<br>*In which country do you live?*	radio, Required<br>1 Switzerland<br>2 Germany<br>3 Austria<br>88 other
19	domicile_88<br>Show the field ONLY if: [domicile] = '88'	residence - other<br>*Please specify.*	text, Required
20	children_nr<br>Show the field ONLY if: [sex] = '1' and [age] >= 18	Number of children	dropdown, Required<br>0 none<br>1 1<br>2 2<br>3 3<br>4 4<br>5 5<br>6 6<br>7 7<br>8 8<br>9 9<br>10 10<br>11 more than 10
21	civil<br>Show the field ONLY if: [sex] = '1' and [age] >= 18	Marital status	dropdown, Required<br>1 single<br>2 married

			3 civil union<br>4 widowed<br>5 divorced<br>88 other /no anwser
22	education_ch<br>education_de<br>education_at<br>education_other	education:<br>Please select your highest educational degree.	radio, Required<br>0 No degree<br>1 Secondary level I (=obligatory school: secondary school, high school)<br>2 Secondary level II general education (general qualification for university entrance)<br>3 Secondary level II vocational education (apprenticeship, higher vocational diploma)<br>4 Tertiary level higher vocational education (higher technical school HF, e.g. HWV, HFG,<br>5 Tertiary level college (university, technical college, teacher training college)<br>88 Other
26	education_88<br>Show the field ONLY if:<br>[education_ch] = '88' or [educati on_de] = '88' or [education_at] = '88' or [education_other] = '88'	education - other<br>*Please specify.*	notes, Required
27	education_0<br>Show the field ONLY if:<br>[education_ch] = '0' or [educatio n_de] = '0' or [education_at] = '0' or [education_other] = '0'	How many years did you go to school?	text (number, Min: 0, Max: 12), Required
28	employment<br>Show the field ONLY if: [sex] = '1' and [age] >= 18	employment status:<br>Please select ALL applicable.	checkbox, Required<br>1 employment___1 Full time (100%)<br>2 employment___2 Part time<br>3 employment___3 Hourly wages<br>4 employment___4 Student / in training<br>5 employment___5 Unpaid internship<br>6 employment___6 Self-employed<br>7 employment___7 Unemployed<br>88 employment___88 Other
29	employment_spec<br>Show the field ONLY if:<br>[employment(2)] = '1'	What is your pensum for your part-time work? [%]	dropdown, Required<br>1 5%<br>2 10%<br>3 15%<br>4 20%<br>5 25%<br>6 30%<br>7 35%<br>8 40%<br>9 45%<br>10 50%<br>11 55%<br>12 60%<br>13 65%<br>14 70%<br>15 75%<br>16 80%<br>17 85%<br>18 90%<br>19 95%
30	employment_88<br>Show the field ONLY if:<br>[employment(88)] = '1'	employment status - other<br>*Please specify.*	notes, Required
31	ethnicity<br>Show the field ONLY if: [sex] = '1' and [age] >= 18	Ethnicity (multiple answers possible)<br>*Please select your ethnicity. If you are not sure select the one that fits best. It is possible to select more than one answer if your parents are from different regions.*	checkbox, Required<br>1 ethnicity___1 White (caucasian/light-skinned)<br>2 ethnicity___2 Mediterranean (France; Portugal; Spain; Italy; Greece; Malta; Cyprus)

			3 ethnicity___3 Hispanic (Americans with Spanish or Latin-American origin)<br>4 ethnicity___4 Middle East (arabic countries, Iran, Turkey)<br>5 ethnicity___5 South-Asian (Afghanistan, Bangladesh, Bhutan, India, Maledives, Nepal, Pakistan, Sri Lanka)<br>6 ethnicity___6 East-Asian (China, Hong Kong, Macau, Taiwan, Japan, North Korea, South Korea, Mongolia)<br>7 ethnicity___7 Southeast-Asian (Brunei, Cambodia, Indonesia, Laos, Malaysia, Myanmar, Philippines, Singapore, Thailand, East Timor, Vietnam)<br>8 ethnicity___8 Aborigines (=Australian natives)<br>9 ethnicity___9 Black<br>88 ethnicity___88 Other
32	ethnicity_88<br>Show the field ONLY if: [ethnicity(88)] = '1'	Ethnicity - Other<br>*Please specify.*	notes, Required
33	ethnicity_spec<br>Show the field ONLY if: [ethnicity(1)] = '1'	What region do you come from?<br>*Please specify your descendance. It is possible to select more than one answer if your parents are from different regions.*	checkbox, Required<br>1 ethnicity_spec___1 Europe<br>2 ethnicity_spec___2 Australia<br>3 ethnicity_spec___3 North-America<br>88 ethnicity_spec___88 Other
34	ethnicity_spec_88<br>Show the field ONLY if: [ethnicity_spec(88)] = '1'	region - other<br>*Please specify.*	notes, Required
35	charakteristika_complete	Section Header: *Form Status*<br>Complete?	dropdown<br>0 Incomplete<br>1 Unverified<br>2 Complete

36	mens_age_know	Section Header: *menstrual cycle profile*<br>Do you know how old you were when you had your first menstruation?	radio, Required<br>1 Age known (approx.)<br>99 Age not known<br>2 no menstuation yet
37	mens_age_nr<br>Show the field ONLY if:<br>[mens_age_know] = '1'	How many years ago did you have your first menstruation? Please subtract your your age when you had your first menstruation from your current age.<br>*Number in years.*	dropdown, Required<br>1 1<br>2 2<br>3 3<br>4 4<br>5 5<br>6 6<br>7 7<br>8 8<br>9 9<br>10 10<br>11 11<br>12 12<br>13 13<br>14 14<br>15 15<br>16 16<br>17 17<br>18 18<br>19 19<br>20 20<br>21 21<br>22 22<br>23 23<br>24 24<br>25 25<br>26 26<br>27 27<br>28 28<br>29 29<br>30 30<br>31 31<br>32 32<br>33 33<br>34 34<br>35 35<br>36 36<br>37 37<br>38 38<br>39 39<br>40 40<br>41 41<br>42 42<br>43 43<br>44 44<br>45 45<br>46 46<br>47 47<br>48 48<br>49 49<br>50 50

38	mens_menopause_yn<br>Show the field ONLY if:<br>[mens_age_know] = '1' or [mens_age_know] = '99'	Did you already reach menopause?<br>*Menopause is defined as minimum 12 months without menstruation in women older than 40 years.*	radio, Required<br>1 Yes<br>0 No
39	mens_menopause_nr<br>Show the field ONLY if:<br>[mens_menopause_yn] = '1' and ([mens_age_know] = '1' or [mens_age_know] = '99')	How many years ago did you have your last menstruation?	text (integer, Min: 1, Max: 60), Required
40	excluded_3<br>Show the field ONLY if:<br>[mens_menopause_yn] = '1'	Unfortunately, you do not fulfill required criteria for this study. Nevertheless, we thank you for your willingness and wish you all the best. Please select «submit». Afterwards you can close the window.	descriptive
41	mens_info1<br>Show the field ONLY if:<br>[mens_menopause_yn] = '0'	Following questions are to assess regularity of your menstrual cycle profile. You will be asked questions to cycle duration and period duration. If you do not know exactly how many days your cycle or period lasts, give your best estimation.<br>- cycle duration = number of days between 1st day of menstruation and 1st day of next mensturation<br>- period duration = number of days bleeding	descriptive
42	mens_info2<br>Show the field ONLY if:<br>[mens_menopause_yn] = '0'	Section Header: *current menstrual cycle profile*<br>Following questions refer to your current period. Think about the last 12 months. If you are using hormonal contraception (pill, hormone spiral, implanon, vaginal contraceptive ring, etc.) or you are currently pregnant you will not be asked futher questions about your current period	descriptive
43	mens_current_contracep_yn<br>Show the field ONLY if:<br>[mens_menopause_yn] = '0'	Do you currently use any hormonal contraceptive method?<br>*E.g. pill, hormone spiral, implanon, vaginal contraceptive ring*	radio, Required<br>1 Yes<br>0 No
44	mens_current_ss_yn<br>Show the field ONLY if:<br>[mens_menopause_yn] = '0'	Are you currently pregnant or do breast-feeding?	radio, Required<br>1 Yes<br>0 No
45	mens_current_regular_yn<br>Show the field ONLY if:<br>([mens_age_know] = '1' or [mens_age_know] = '99') and [mens_menopause_yn] = '0' and [mens_current_contracep_yn] = '0' and	Is your cycle duration always more or less regular (+/- 7 days)?<br>*E.g. cycle-duration in April 27 days, in May 33 days, difference = 6days -> YES*	radio, Required<br>1 Yes<br>0 No
46	mens_current_reg_cycle_nr<br>Show the field ONLY if:<br>[mens_current_regular_yn] = '1'	How long does your cycle last on avarage? [days]<br>*Number of days between 1st day of menstruation and 1st day of next menstruation. Mean in normal population is 28days.*	text (integer, Min: 2), Required
47	mens_current_reg_period_nr<br>Show the field ONLY if:<br>[mens_current_regular_yn] = '1'	How long does your period last on avarage? [days]<br>*Days you are bleeding.*	text (integer, Min: 1, Max: 30), Required
48	mens_current_irreg_spec<br>Show the field ONLY if:<br>[mens_current_regular_yn] = '0'	In what way isn't your cycle more or less regular?	dropdown, Required<br>1 no period<br>2 fluctuation >8days<br>88 other
49	mens_current_irreg_none<br>Show the field ONLY if:<br>[mens_current_irreg_spec] = '1'	How many months ago did you have your last menstruation? [months]<br>*Number in months. If it is more than one year please multiply years by 12.*	text (integer, Min: 1), Required
50	mens_current_irreg_min<br>Show the field ONLY if:<br>[mens_current_irreg_spec] = '2'	What has been your shortest cycle time during the last 12 months? [days]<br>*Number of days between 1st day of menstruation and 1st day of next menstruation*	text (integer, Min: 1), Required
51	mens_current_irreg_max<br>Show the field ONLY if:<br>[mens_current_irreg_spec] = '2'	What has been your longest cycle time during the last 12 months? [days]<br>*Number of days between 1st day of menstruation and 1st day of next menstruation*	text (integer, Min: 1), Required
52	mens_current_irreg_spec_88<br>Show the field ONLY if:<br>[mens_current_irreg_spec] = '88'	Other irregularities<br>*Please specify.*	notes, Required
53	mens_current_irreg_period_nr<br>Show the field ONLY if:<br>[mens_current_irreg_spec] = '2' or [mens_current_irreg_spec] = '88'	How long does your period last on avarage? [days]<br>*Days you are bleeding.*	text (integer, Min: 1), Required
54	mens_current_irreg_cyclenr<br>Show the field ONLY if:<br>[mens_current_irreg_spec] = "2" or [mens_current_irreg_spec] = "88"	How many menstrual cycles do you have on average per year?	text (integer, Min: 2, Max: 30), Required

55	mens_info3<br>Show the field ONLY if:<br>[mens_menopause_yn] = '0'	Section Header: *menstural cycle profile in the past*<br>Following questions refer to your period in the past. Think about periods more than 12 months ago. We are interested in your natural menstural cycle profile. Thus do not include the first year of having menstruations. Irregularities are normal in this period and should not be declared here. Also you should not include times of pregnancy, breast-feeding or hormonal contraception. If you have used hormonal contraception (pill, hormone spiral, implanon, vaginal contraceptive ring, etc.) please answer this questions for the time before you started using it	descriptive
56	mens_cycle90<br>Show the field ONLY if:<br>([mens_age_know] = '1' or [mens_age_know] = '99') and [mens_menopause_yn] = '0'	Have you ever had a cycle duration of more than 90 days?	radio, Required<br>1 Yes<br>0 No
57	mens_past_regular_yn<br>Show the field ONLY if:<br>([mens_age_know] = '1' or [mens_age_know] = '99') and [mens_menopause_yn] = '0'	Has your cycle duration always been more or less regular (+/- 7 days)?<br>*E.g. cycle-duration in April 27 days, in May 33 days, difference = 6days -> YES?*	radio, Required<br>1 Yes<br>0 No
58	mens_past_reg_nr<br>Show the field ONLY if:<br>[mens_current_regular_yn] = '0' and [mens_past_regular_yn] = '1'	For how many years have your cycle duration been regular? [years]<br>*A long guess is sufficient.*	text (integer, Min: 1), Required
59	mens_past_reg_irreg_nr<br>Show the field ONLY if:<br>[mens_current_regular_yn] = '0' and [mens_past_regular_yn] = '1'	For how many years do you already have irregular cycle durations? [years]<br>*A long guess is sufficient.*	text (integer, Min: 1), Required
60	mens_past_reg_cycle_nr<br>Show the field ONLY if:<br>[mens_past_regular_yn] = '1'	How long did your cycle last on avarage (approx.)? [days]<br>*Number of days between 1st day of menstruation and 1st day of next menstruation. Mean in normal population is 28days.*	text (integer, Min: 3), Required
61	mens_past_reg_period_nr<br>Show the field ONLY if:<br>[mens_past_regular_yn] ="1"	How long did your period last on avarage? [days]<br>*Days you were bleeding.*	text (integer, Min: 1), Required
62	mens_past_irreg_nr<br>Show the field ONLY if:<br>[mens_current_regular_yn] = '1' and [mens_past_regular_yn] = '0'	For how many years have you had irregular cycle durations? [years]	text (integer, Min: 1), Required
63	mens_past_irreg_reg_nr<br>Show the field ONLY if:<br>[mens_current_regular_yn] = '1' and [mens_past_regular_yn] = '0'	For how many years do you now have regular cycle durations? [years]	text (number, Min: 1), Required
64	mens_past_irreg_spec<br>Show the field ONLY if:<br>[mens_past_regular_yn] = '0'	In what way hasn't your cycle been more or less regular?	dropdown, Required<br>1 no period<br>2 fluctuation >8days<br>88 other
65	mens_past_irreg_none<br>Show the field ONLY if:<br>[mens_past_irreg_spec] = '1'	How many months ago did you have your last menstruation? [months]<br>*Number in months. If it is more than one year please multiply years by 12.*	text (integer, Min: 1), Required
66	mens_past_irreg_min<br>Show the field ONLY if:<br>[mens_past_irreg_spec] = '2'	What has been your shortest cycle time you remember? [days]<br>*Number of days between 1st day of menstruation and 1st day of next menstruation*	text (integer, Min: 3), Required
67	mens_past_irreg_max<br>Show the field ONLY if:<br>[mens_past_irreg_spec] = '2'	What has been your longest cycle time you remember? [days]<br>*Number of days between 1st day of menstruation and 1st day of next menstruation*	text (integer, Min: 3), Required
68	mens_past_irrec_spec_88<br>Show the field ONLY if:<br>[mens_past_irreg_spec] = '88'	Other irregularities<br>*Please specify.*	notes, Required
69	mens_past_irreg_period_nr<br>Show the field ONLY if:<br>[mens_past_irreg_spec] = '2' or [mens_past_irreg_spec] = '88'	How long did your period last on avarage? [days]<br>*Days you are bleeding.*	text (integer, Min: 1), Required
70	mens_past_irreg_cyclenr<br>Show the field ONLY if:<br>[mens_past_irreg_spec] = "2" or [mens_past_irreg_spec] = "88"	How many menstrual cycles did you have on average per year?	text (integer, Min: 2, Max: 30), Required
71	mens_thelarche<br>Show the field ONLY if:<br>[mens_age_know] = '2'	Is the the onset of your breast development more than 3 years ago?	radio, Required<br>1 Yes<br>0 No

72	irregular<br>Show the field ONLY if:<br>([mens_age_know] = '2') or ([me ns_age_know] = '1' and ([mens_a ge_nr] = 1 or [mens_age_nr] = 2 or [mens_age_nr] = 3)) or ((([me ns_age_know] = '1' and [mens_a ge_nr] > 3) or ([mens_age_know] = '99')) and (([mens_current_reg ular_yn] = '1' and ([mens_curren t_reg_cycle_nr] < 21 or [mens_cu rrent_reg_cycle_nr] > 35)) or ([m ens_cycle90] = '1') or ([mens_cur rent_regular_yn] = '0' and (([men s_current_irreg_spec] = '1' and [mens_current_irreg_none] >= 3) or ([mens_current_irreg_spec] = '2' and ([mens_current_irreg_mi n] < 21 or [mens_current_irreg_ max] >35 or [mens_current_irre g_cyclenr] < 8)) or ([mens_curren t_irreg_spec] = '88' and [mens_c urrent_irreg_cyclenr] < 8))) or ([mens_past_regular_yn] = '1' an d ([mens_past_reg_cycle_nr] < 2 1 or [mens_past_reg_cycle_nr] > 35)) or ([mens_past_regular_yn] = '0' and (([mens_past_irreg_spe c] = '1' and [mens_past_irreg_no ne] >=3) or ([mens_past_irreg_sp ec] = '2' and ([mens_past_irreg_ min] < 21 OR [mens_past_irreg_ max] >35 or [mens_past_irreg_c yclenr] < 8)) or ([mens_past_irre g_spec] = '88' and	Your menstrual cycle profile is regarded irregular.	radio<br>Field Annotation: @HIDDEN-SURVEY
73	diagnosekriterien_teil_1_comple te	Section Header: *Form Status*<br>Complete?	dropdown<br>0 Incomplete<br>1 Unverified<br>2 Complete

Instrument: **diagnostic criteria - part 2** (diagnosekriterien_teil_2) Enabled as survey

74	clhy_acne_yn	Section Header: *clinical hyperandrogenism*<br>Do you have or did you ever have acne?	radio, Required<br>1 Yes<br>0 No<br>99 Unknown
75	clhy_alopezia_yn	Do you have or did you ever have exessive hair loss?<br>*To answer take a look at following picture.*	radio, Required<br>1 Yes<br>0 No<br>99 Unknown
76	clhy_alopezia_pic	To illustrate "excessive hair loss" you are shown following picture with different grades of hair loss. If one of these pictures suits you please answer "YES" in the question above.<br>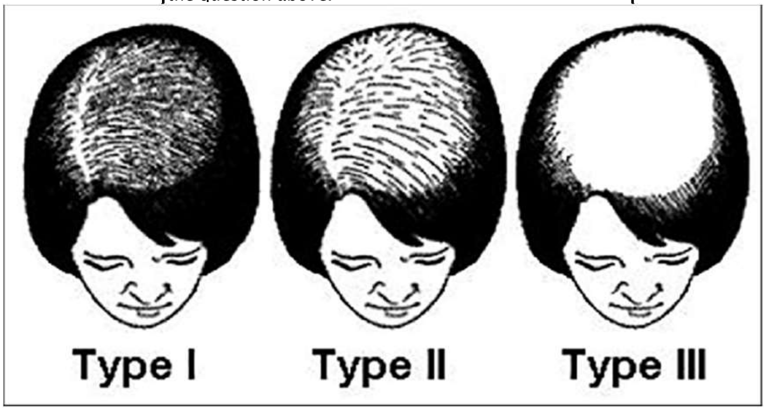	descriptive
77	clhy_alopezia_quant<br>Show the field ONLY if:<br>[clhy_alopezia_yn] = '1'	Take a look at the different grades of hair loss illustrated in the picture. What grade would you classify yourself?	dropdown, Required<br>1 Type 1<br>2 Type 2<br>3 Type 3
78	clhy_hirsutism_yn	Do you have or did you ever have excess hair-growth compared to other women?<br>*To answer take a look at following picture.*	radio, Required<br>1 Yes<br>0 No<br>99 Unknown
79	clhy_hirsutism_pic	To illustrate "excess hair-growth" you are shown following picture with different grades of hair-growth. If one of these pictures suits, even if it is only one body region, you please answer "YES" in the question above.<br>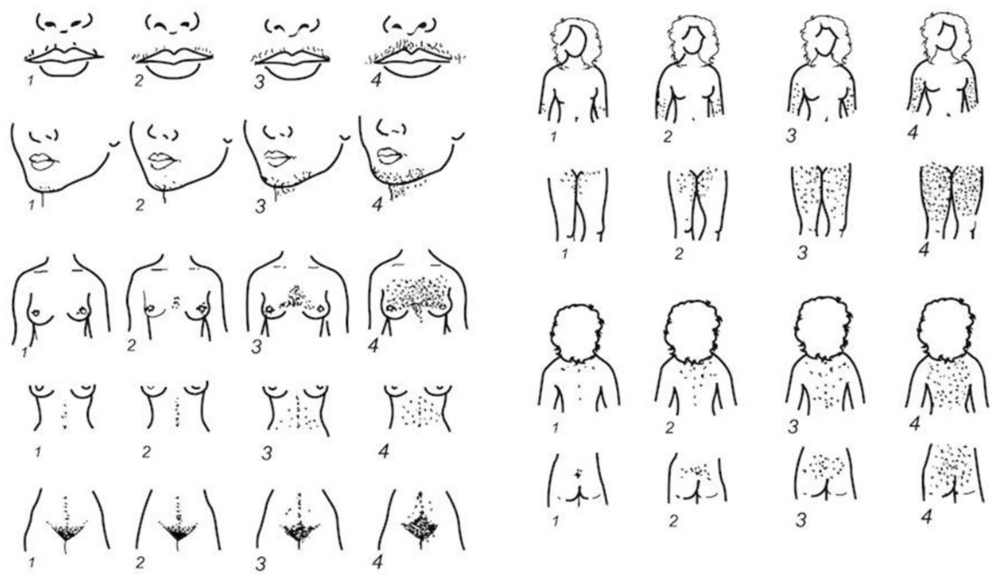	descriptive
80	clhy_hirsutism_spec<br>Show the field ONLY if:<br>[clhy_hirsutism_yn] = '1'	What body regions are affected?	checkbox, Required<br>1 clhy_hirsutism_spec___1 face<br>2 clhy_hirsutism_spec___2 breast

			3 | clhy_hirsutism_spec___3 | upper abdomen (breast to belly button, left row<br>4 | clhy_hirsutism_spec___4 | lower abdomen (belly botton to bikini line, left<br>5 | clhy_hirsutism_spec___5 | upper arm<br>6 | clhy_hirsutism_spec___6 | tight<br>7 | clhy_hirsutism_spec___7 | upper back (neck to waist, right row 3)<br>8 | clhy_hirsutism_spec___8 | lower back (waist to buttocks, right row 4)<br>88 | clhy_hirsutism_spec___88 | other
81	clhy_hirsutism_spec_88<br>Show the field ONLY if:<br>[clhy_hirsutism_spec(88)] = '1'	body regions - other<br>*Please specify.*	notes, Required
82	clhy_hirsutism_spec_face<br>Show the field ONLY if:<br>[clhy_hirsutism_spec(1)] = '1'	Where in your face?	checkbox, Required<br>1 | clhy_hirsutism_spec_face 1 | upper lips<br>2 | clhy_hirsutism_spec_face 2 | chin<br>88 | clhy_hirsutism_spec_face 88 | other
83	clhy_hirsutism_spec_face_88<br>Show the field ONLY if:<br>[clhy_hirsutism_spec_face(88)] = '1'	face - other<br>*Please specify.*	notes, Required
84	clhy_hirsutism_quant1_1<br>Show the field ONLY if:<br>[clhy_hirsutism_spec_face(1)] = '1'	Take a look at the different grades of hair-growth illustrated in the picture. What grade would you classify yourself regarding your upper lips?	dropdown, Required<br>1 | 1<br>2 | 2<br>3 | 3<br>4 | 4
85	clhy_hirsutism_quant1_2<br>Show the field ONLY if:<br>[clhy_hirsutism_spec_face(2)] = '1'	Take a look at the different grades of hair-growth illustrated in the picture. What grade would you classify yourself regarding your chin?	dropdown, Required<br>1 | 1<br>2 | 2<br>3 | 3<br>4 | 4
86	clhy_hirsutism_quant1_88<br>Show the field ONLY if:<br>[clhy_hirsutism_spec_face(88)] = '1'	Take a look at the different grades of hair-growth illustrated in the picture. What grade would you classify yourself regarding your other face-region?	dropdown, Required<br>1 | 1<br>2 | 2<br>3 | 3<br>4 | 4
87	clhy_hirsutism_quant2<br>Show the field ONLY if:<br>[clhy_hirsutism_spec(2)] = '1'	Take a look at the different grades of hair-growth illustrated in the picture. What grade would you classify yourself regarding your breast?	dropdown, Required<br>1 | 1<br>2 | 2<br>3 | 3<br>4 | 4
88	clhy_hirsutism_quant3<br>Show the field ONLY if:<br>[clhy_hirsutism_spec(3)] = '1'	Take a look at the different grades of hair-growth illustrated in the picture. What grade would you classify yourself regarding your upper abdomen?	dropdown, Required<br>1 | 1<br>2 | 2<br>3 | 3<br>4 | 4
89	clhy_hirsutism_quant4<br>Show the field ONLY if:<br>[clhy_hirsutism_spec(4)] = '1'	Take a look at the different grades of hair-growth illustrated in the picture. What grade would you classify yourself regarding your lower abdomen?	dropdown, Required<br>1 | 1<br>2 | 2<br>3 | 3<br>4 | 4
90	clhy_hirsutism_quant5<br>Show the field ONLY if:<br>[clhy_hirsutism_spec(5)] = '1'	Take a look at the different grades of hair-growth illustrated in the picture. What grade would you classify yourself regarding your upper arms?	dropdown, Required<br>1 | 1<br>2 | 2<br>3 | 3<br>4 | 4
91	clhy_hirsutism_quant6<br>Show the field ONLY if:<br>[clhy_hirsutism_spec(6)] = '1'	Take a look at the different grades of hair-growth illustrated in the picture. What grade would you classify yourself regarding your tights?	dropdown, Required<br>1 | 1<br>2 | 2<br>3 | 3

			4 = 4
92	clhy_hirsutism_quant7<br>Show the field ONLY if:<br>[clhy_hirsutism_spec(7)] = '1'	Take a look at the different grades of hair-growth illustrated in the picture. What grade would you classify yourself regarding your upper back?	dropdown, Required<br>1 = 1<br>2 = 2<br>3 = 3<br>4 = 4
93	clhy_hirsutism_quant8<br>Show the field ONLY if:<br>[clhy_hirsutism_spec(8)] = '1'	Take a look at the different grades of hair-growth illustrated in the picture. What grade would you classify yourself regarding your lower back?	dropdown, Required<br>1 = 1<br>2 = 2<br>3 = 3<br>4 = 4
94	clhy_hirsutism_quant88<br>Show the field ONLY if:<br>[clhy_hirsutism_spec(88)] = '1'	Take a look at the different grades of hair-growth illustrated in the picture. What grade would you classify yourself regarding your other body-region (as described)?	dropdown, Required<br>1 = 1<br>2 = 2<br>3 = 3<br>4 = 4
95	cl_hyperandrogenism<br>Show the field ONLY if:<br>[clhy_acne_yn] = '1' or [clhy_alopezia_yn] = '1' or [clhy_hirsutism_yn] = '1'	clinical hyperandrogenism	radio<br>Field Annotation: @HIDDEN-SURVEY
96	bchy_hormone_yn	Section Header: *biochemical hyperandrogenism*<br>Has there ever been done blood examination for female and male sex hormones?	radio, Required<br>1 Yes<br>0 No<br>99 Unknown
97	bchy_hormone_m_yn<br>Show the field ONLY if:<br>[bchy_hormone_yn] = '1'	Thus, have there been found elevated levels of male sex hormones?	radio, Required<br>1 Yes<br>0 No<br>99 Unknown
98	bc_hyperandrogenism<br>Show the field ONLY if:<br>[bchy_hormone_m_yn] = '1'	biochemical hyperandrogenism	radio<br>Field Annotation: @HIDDEN-SURVEY
99	pcom_us_yn	Section Header: *morphological polycystic ovaries*<br>Has there ever been done a vaginal sonography?<br>*For this purpose there is insertet a slender, rod-shaped receiving transducer into your vagina.*	radio, Required<br>1 Yes<br>0 No<br>99 Unknown
100	pcom_us_ovarien_yn<br>Show the field ONLY if:<br>[pcom_us_yn] = '1'	Thus, have there been detected an increased ovary size?	radio, Required<br>1 Yes<br>0 No<br>99 Unknown
101	pcom_us_cysts_yn<br>Show the field ONLY if:<br>[pcom_us_yn] = '1'	Thus, have there been detected an increased number of follicles (sometimes called cysts)?<br>*These are "bubbles" inside the ovary that occur when immature egg cells are accumulating.*	radio, Required<br>1 Yes<br>0 No<br>99 Unknown
102	pcom<br>Show the field ONLY if:<br>[pcom_us_cysts_yn] = '1' or [pcom_us_ovarien_yn] = '1'	morphological polycystic ovaries	radio<br>Field Annotation: @HIDDEN-SURVEY
103	pcos_diagnosis	Section Header: *received diagnosis*<br>Did your gynecologist tell you that you have polycystic ovary syndrome (PCOS)?	radio, Required<br>1 Yes<br>0 No<br>99 Unknown
104	pcos_dd_ags	Section Header: *differentialdiagnoses*<br>Have you been diagnosed with adrenogenital syndrome?<br>*Falls Sie nicht wissen was das ist, kreuzen sie "Nein/Unbekannt" an.*	radio, Required<br>1 Yes<br>0 No / Unknown
105	pcos_dd_prolaktinom	Have you been diagnosed with a prolactinoma?<br>*Prolactinoma is a benign tumor of the pituary gland and is producing the hormone prolactin.*	radio, Required<br>1 Yes<br>0 No / Unknown

106	pcos_dd<br>Show the field ONLY if:<br>[pcos_dd_ags] = '1' or [pcos_dd_prolaktinom] = '1'	fulfill differentialdiagnosis	descriptive<br>Field Annotation: @HIDDEN-SURVEY
107	excluded_4<br>Show the field ONLY if:<br>[pcos_dd_prolaktinom] = '1' or [pcos_dd_ags] = '1'	Unfortunately, you do not fulfill required criteria for this study. Nevertheless, we thank you for your willingness and wish you all the best. Please select «submit». Afterwards you can close the window.	descriptive
108	diagnosekriterien_teil_2_complete	Section Header: *Form Status*<br>Complete?	dropdown<br>0 Incomplete<br>1 Unverified<br>2 Complete

Programing: only elegible participants can continue

([pcos_diagnosis] or (((([mens_age_know] = '2') **or** ([mens_age_know] = '1' and ([mens_age_nr] = 1 or [mens_age_nr] = 2 or [mens_age_nr] = 3)) **or** ((([mens_age_know] = '1' and [mens_age_nr] > 3) or ([mens_age_know] = '99')) and ((([mens_current_regular_yn] = '1' and ([mens_current_reg_cycle_nr] < 21 or [mens_current_reg_cycle_nr] > 35)) **or** ([mens_cycle90] = '1') **or** ([mens_current_regular_yn] = '0' and ((([mens_current_irreg_spec] = '1' and [mens_current_irreg_none] >= 3) or ([mens_current_irreg_spec] = '2' and ([mens_current_irreg_min] < 21 or [mens_current_irreg_max] >35 or [mens_current_irreg_cyclenr] < 8)) or ([mens_current_irreg_spec] = '88' and [mens_current_irreg_cyclenr] <8))) **or** ([mens_past_regular_yn] = '1' and ([mens_past_reg_cycle_nr] < 21 or [mens_past_reg_cycle_nr] > 35)) **or** ([mens_past_regular_yn] = '0' and ((([mens_past_irreg_spec] = '1' and [mens_past_irreg_none] >=3) or ([mens_past_irreg_spec] = '2' and ([mens_past_irreg_min] < 21 OR [mens_past_irreg_max] >35 or [mens_past_irreg_cyclenr] < 8)) or ([mens_past_irreg_spec] = '88' and [mens_past_irreg_cyclenr] < 8)))))) and ([clhy_acne_yn] = '1' or [clhy_alopezia_yn] = '1' or [clhy_hirsutism_yn] = '1')) or ((([mens_age_know] = '2') **or** ([mens_age_know] = '1' and ([mens_age_nr] = 1 or [mens_age_nr] = 2 or [mens_age_nr] = 3)) **or** ((([mens_age_know] = '1' and [mens_age_nr] > 3) or ([mens_age_know] = '99')) and ((([mens_current_regular_yn] = '1' and ([mens_current_reg_cycle_nr] < 21 or [mens_current_reg_cycle_nr] > 35)) **or** ([mens_cycle90] = '1') **or** ([mens_current_regular_yn] = '0' and ((([mens_current_irreg_spec] = '1' and [mens_current_irreg_none] >= 3) or ([mens_current_irreg_spec] = '2' and ([mens_current_irreg_min] < 21 or [mens_current_irreg_max] >35 or [mens_current_irreg_cyclenr] < 8)) or ([mens_current_irreg_spec] = '88' and [mens_current_irreg_cyclenr] <8))) **or** ([mens_past_regular_yn] = '1' and ([mens_past_reg_cycle_nr] < 21 or [mens_past_reg_cycle_nr] > 35)) **or** ([mens_past_regular_yn] = '0' and ((([mens_past_irreg_spec] = '1' and [mens_past_irreg_none] >=3) or ([mens_past_irreg_spec] = '2' and ([mens_past_irreg_min] < 21 OR [mens_past_irreg_max] >35 or [mens_past_irreg_cyclenr] < 8)) or ([mens_past_irreg_spec] = '88' and [mens_past_irreg_cyclenr] < 8)))))) and ([bchy_hormone_m_yn] = '1')) or ((([mens_age_know] = '2') **or** ([mens_age_know] = '1' and ([mens_age_nr] = 1 or [mens_age_nr] = 2 or [mens_age_nr] = 3)) **or** ((([mens_age_know] = '1' and [mens_age_nr] > 3) or ([mens_age_know] = '99')) and ((([mens_current_regular_yn] = '1' and ([mens_current_reg_cycle_nr] < 21 or [mens_current_reg_cycle_nr] > 35)) **or** ([mens_cycle90] = '1') **or** ([mens_current_regular_yn] = '0' and ((([mens_current_irreg_spec] = '1' and [mens_current_irreg_none] >= 3) or ([mens_current_irreg_spec] = '2' and ([mens_current_irreg_min] < 21 or [mens_current_irreg_max] >35 or [mens_current_irreg_cyclenr] < 8)) or ([mens_current_irreg_spec] = '88' and [mens_current_irreg_cyclenr] <8))) **or** ([mens_past_regular_yn] = '1' and ([mens_past_reg_cycle_nr] < 21 or [mens_past_reg_cycle_nr] > 35)) **or** ([mens_past_regular_yn] = '0' and ((([mens_past_irreg_spec] = '1' and [mens_past_irreg_none] >=3) or ([mens_past_irreg_spec] = '2' and ([mens_past_irreg_min] < 21 OR [mens_past_irreg_max] >35 or [mens_past_irreg_cyclenr] < 8)) or ([mens_past_irreg_spec] = '88' and [mens_past_irreg_cyclenr] < 8)))))) and ([pcom_us_cysts_yn] = '1' or [pcom_us_ovarien_yn] = '1')) or ((([clhy_acne_yn] = '1' or [clhy_alopezia_yn] = '1' or [clhy_hirsutism_yn] = '1') and ([pcom_us_cysts_yn] = '1' or [pcom_us_ovarien_yn] = '1')) or (([bchy_hormone_m_yn] = '1') and ([pcom_us_cysts_yn] = '1' or [pcom_us_ovarien_yn] = '1')))) and ([pcos_dd_ags] = '0' and [pcos_dd_prolaktinom] = '0')

109	pcos_info<br>Show the field ONLY if:<br>[pcos_diagnosis] = '0'	You stated that you haven't received any former diagnosis for PCOS yet. According to your answers you fulfill criteria for PCOS. Please contact your gynecologist for further assessment. Inform him/her that you have been participating in an online survey telling you that you fulfill criteria for PCOS. For further inquiry we are at his/her disposal (julia.estermann@students.unibe.ch).	descriptive
110	cosm_neg_yn	Has your cosmetic appearance changed negatively due to PCOS?	radio, Required<br>1 Yes<br>0 No
111	cosm_probl	Please select the cosmetic issues affecting you in the present or past.<br>*Some of the issues have been investigated earlier in this survey. Please check it again here. There will be asked some more detailed questions about those issues.*	checkbox, Required<br>1 cosm_probl___1 acne<br>2 cosm_probl___2 alopecia<br>3 cosm_probl___3 excess hair growth<br>4 cosm_probl___4 overweight<br>88 cosm_probl___88 other<br>0 cosm_probl___0 none<br>Field Annotation: @NONEOFTHEABOVE=0
112	cosm_probl_88<br>Show the field ONLY if:<br>[cosm_probl(88)] = '1'	What other cosmetic issues?<br>*Please specify.*	notes, Required
113	cosm_probl_acne_info<br>Show the field ONLY if:<br>[clhy_acne_yn] = '1'	Section Header: *acne*<br>Earlier in this survey you stated to be affected by acne. Please answer following questions about it.	descriptive
114	cosm_probl_acne_bother_yn<br>Show the field ONLY if:<br>[cosm_probl(1)] = '1' or [clhy_acne_yn] = '1'	Does/did your acne bother you?	radio, Required<br>1 Yes<br>0 No
115	cosm_probl_acne_adv_yn<br>Show the field ONLY if:<br>[cosm_probl(1)] = '1' or [clhy_acne_yn] = '1'	Have you been consultet by your gynecologists about this?	radio, Required<br>1 Yes<br>0 No
116	cosm_probl_acne_adv_sc<br>Show the field ONLY if:<br>[cosm_probl_acne_adv_yn] = '1'	Please rate your satisfaction with consultation regarding acne?<br>*Drag the blue slider and drop it at the desired position.*	slider (number), Required<br>Slider labels: 0, 50, 100<br>Custom alignment: RH
117	cosm_probl_acne_adv_wish<br>Show the field ONLY if:<br>[cosm_probl_acne_adv_yn] = '0'	Would you have wished for a consultation by your gynecologist regarding acne?	radio, Required<br>1 Yes<br>0 No
118	cosm_probl_acne_th_yn<br>Show the field ONLY if:<br>[cosm_probl(1)] = '1' or [clhy_acne_yn] = '1'	Have you tried any therapy for acne?	radio, Required<br>1 Yes<br>0 No<br>99 Unknown
119	cosm_probl_acne_th_spec<br>Show the field ONLY if:<br>[cosm_probl_acne_th_yn] = '1'	Which therapy method have you tried already?<br>*If you are not sure about one of the options you can check it to have a look at the sub-categories. If there isn't anything suitable you can unckeck the option again.*	checkbox, Required<br>1 cosm_probl_acne_th_spec___1 lifestyle intervention (diet, exercise, etc.)<br>2 cosm_probl_acne_th_spec___2 medication (including contraceptive pill)<br>3 cosm_probl_acne_th_spec___3 psychotherapy<br>4 cosm_probl_acne_th_spec___4 bariatric surgery (e.g. gastric bypass)<br>88 cosm_probl_acne_th_spec___88 other
120	cosm_probl_acne_th_spec_88<br>Show the field ONLY if:<br>[cosm_probl_acne_th_spec(88)] = '1'	What other therapeutic options?<br>*Please specify.*	notes, Required
121	cosm_probl_acne_th1_spec<br>Show the field ONLY if:<br>[cosm_probl_acne_th_spec(1)] = '1'	Which lifestyle interventions have you tried?	checkbox, Required<br>1 cosm_probl_acne_th1_spec___1 Behavioural interventions (e.g. goal-setting, self-monitoring, slower eating, etc.)<br>2 cosm_probl_acne_th1_spec___2 attitude<br>3 cosm_probl_acne_th1_spec___3 dietary interventions (e.g. well-balanced, reduce energy intake, etc.)<br>4 cosm_probl_acne_th1_spec___4 physical activity

			5 cosm_probl_acne_th1_spec___5 weight assessment and reduction<br>88 cosm_probl_acne_th1_spec___88 other
122	cosm_probl_acne_th1_spec_88<br>Show the field ONLY if:<br>[cosm_probl_acne_th1_spec(88)] = '1'	What other lifestyle intervention?<br>*Please specify.*	notes, Required
123	cosm_probl_acne_th2_spec<br>Show the field ONLY if:<br>[cosm_probl_acne_th_spec(2)] = '1'	Please select the applicable medications:	checkbox, Required<br>1 cosm_probl_acne_th2_spec___1 contraceptive pill<br>2 cosm_probl_acne_th2_spec___2 anti-androgens<br>3 cosm_probl_acne_th2_spec___3 metformin<br>88 cosm_probl_acne_th2_spec___88 other
124	cosm_probl_acne_th2_spec_88<br>Show the field ONLY if:<br>[cosm_probl_acne_th2_spec(88)] = '1'	What other medication?<br>*Please specify.*	notes, Required
125	cosm_probl_acne_th_effect<br>Show the field ONLY if:<br>[cosm_probl_acne_th_yn] = '1'	Were the therapy attempts effective?	dropdown, Required<br>1 Yes, completely<br>2 Yes, partially<br>0 No, not at all
126	cosm_probl_acne_th_act<br>Show the field ONLY if:<br>[cosm_probl_acne_th_yn] = '1'	Which of the therapy methods are you still currently implementing?	checkbox, Required<br>1 cosm_probl_acne_th_act___1 lifestyle intervention (diet, exercise, etc.)<br>2 cosm_probl_acne_th_act___2 medication (including contraceptive pill)<br>3 cosm_probl_acne_th_act___3 psychotherapy<br>4 cosm_probl_acne_th_act___4 bariatric surgery (e.g. gastric bypass)<br>88 cosm_probl_acne_th_act___88 other<br>0 cosm_probl_acne_th_act___0 no therapy anymore<br>Field Annotation: @NONEOFTHEABOVE=0
127	cosm_probl_acne_th1_act<br>Show the field ONLY if:<br>[cosm_probl_acne_th_act(1)] = '1'	Please select the lifestyle interventions you are still currently performing:	checkbox, Required<br>1 cosm_probl_acne_th1_act___1 Behavioural interventions (e.g. goal-setting, self-monitoring, slower eating, etc.)<br>2 cosm_probl_acne_th1_act___2 attitude<br>3 cosm_probl_acne_th1_act___3 dietary interventions (e.g. well-balanced, reduce energy intake, etc.)<br>4 cosm_probl_acne_th1_act___4 physical activity<br>5 cosm_probl_acne_th1_act___5 weight assessment and reduction<br>88 cosm_probl_acne_th1_act___88 other (as described above)
128	cosm_probl_acne_th2_act<br>Show the field ONLY if:<br>[cosm_probl_acne_th_act(2)] = '1'	Please select the medication you are still currently taking:	checkbox, Required<br>1 cosm_probl_acne_th2_act___1 contraceptive pill<br>2 cosm_probl_acne_th2_act___2 anti-androgens<br>3 cosm_probl_acne_th2_act___3 metformin<br>88 cosm_probl_acne_th2_act___88 other (as described above)
129	cosm_probl_acne_th_adv_yn<br>Show the field ONLY if:<br>[cosm_probl_acne_th_yn] = '1'	Have you been consulted by your gynecologist regarding the therapy attempts?	radio, Required<br>1 Yes<br>0 No
130	cosm_probl_acne_th_adv_sc<br>Show the field ONLY if:<br>[cosm_probl_acne_th_adv_yn] = '1'	Please rate your satisfaction with consultation regarding therapy attempts.<br>*Drag the blue slider and drop it at the desired position.*	slider (number), Required<br>Slider labels: 0, 50, 100<br>Custom alignment: RH
131	cosm_probl_acne_th_adv_wish<br>Show the field ONLY if:<br>[cosm_probl_acne_th_adv_yn] = '0'	Would you have wished for a consultation by your gynecologist regarding therapy attempts?	radio, Required<br>1 Yes<br>0 No

132	cosm_probl_alop_info<br>Show the field ONLY if:<br>[clhy_alopezia_yn] = '1'	Section Header: *alopecia*<br>Earlier in this survey you stated to be affected by alopecia. Please answer following questions about it.	descriptive
133	cosm_probl_alop_bother_yn<br>Show the field ONLY if:<br>[cosm_probl(2)] = '1' or<br>[clhy_alopezia_yn] = '1'	Does/did your alopecia bother you?	radio, Required<br>1 Yes<br>0 No
134	cosm_probl_alop_adv_yn<br>Show the field ONLY if:<br>[cosm_probl(2)] = '1' or<br>[clhy_alopezia_yn] = '1'	Have you been consultet by your gynecologists about this?	radio, Required<br>1 Yes<br>0 No
135	cosm_probl_alop_adv_sc<br>Show the field ONLY if:<br>[cosm_probl_alop_adv_yn] = '1'	Please rate your satisfaction with consultation regarding alopecia?<br>*Drag the blue slider and drop it at the desired position.*	slider (number), Required<br>Slider labels: 0, 50, 100<br>Custom alignment: RH
136	cosm_probl_alop_adv_wish<br>Show the field ONLY if:<br>[cosm_probl_alop_adv_yn] = '0'	Would you have wished for a consultation by your gynecologist regarding alopecia?	radio, Required<br>1 Yes<br>0 No
137	cosm_probl_alop_th_yn<br>Show the field ONLY if:<br>[cosm_probl(2)] = '1' or<br>[clhy_alopezia_yn] = '1'	Have you tried any therapy for alopecia?	radio, Required<br>1 Yes<br>0 No<br>99 Unknown
138	cosm_probl_alopecia_th_spec<br>Show the field ONLY if:<br>[cosm_probl_alopecia_th_yn] = '1'	Which therapy method have you tried already?<br>*If you are not sure about one of the options you can check it to have a look at the sub-categories. If there isn't anything suitable you can unckeck the option again.*	checkbox, Required<br>1 cosm_probl_alop_th_spec 1 lifestyle intervention (diet, exercise, etc.)<br>2 cosm_probl_alop_th_spec 2 medication (including contraceptive pill)<br>3 cosm_probl_alop_th_spec 3 psychotherapy<br>4 cosm_probl_alop_th_spec 4 bariatric surgery (e.g. gastric bypass)<br>88 cosm_probl_alop_th_spec 88 other
139	cosm_probl_alop_th_spec_88<br>Show the field ONLY if:<br>[cosm_probl_alop_th_spec(88)] = '1'	What other therapeutic options?<br>*Please specify.*	notes, Required
140	cosm_probl_alop_th1_spec<br>Show the field ONLY if:<br>[cosm_probl_alop_th_spec(1)] = '1'	Which lifestyle interventions have you tried?	checkbox, Required<br>1 cosm_probl_alop_th1_spec 1 Behavioural interventions (e.g. goal-setting, self-monitoring, slower eating, etc.)<br>2 cosm_probl_alop_th1_spec 2 attitude<br>3 cosm_probl_alop_th1_spec 3 dietary interventions (e.g. well-balanced, reduce energy intake, etc.)<br>4 cosm_probl_alop_th1_spec 4 physical activity<br>5 cosm_probl_alop_th1_spec 5 weight assessment and reduction<br>88 cosm_probl_alop_th1_spec 88 other
141	cosm_probl_alop_th1_spec_88<br>Show the field ONLY if:<br>[cosm_probl_alop_th1_spec(88)] = '1'	What other lifestyle intervention?<br>*Please specify.*	notes, Required
142	cosm_probl_alop_th2_spec<br>Show the field ONLY if:<br>[cosm_probl_alop_th_spec(2)] = '1'	Please select the applicable medications:	checkbox, Required<br>1 cosm_probl_alop_th2_spec 1 contraceptive pill<br>2 cosm_probl_alop_th2_spec 2 anti-androgens<br>3 cosm_probl_alop_th2_spec 3 metformin<br>88 cosm_probl_alop_th2_spec 88 other
143	cosm_probl_alop_th2_spec_88<br>Show the field ONLY if:<br>[cosm_probl_alop_th2_spec(88)] = '1'	What other medication?<br>*Please specify.*	notes, Required
144	cosm_probl_alop_th_effect<br>Show the field ONLY if:<br>[cosm_probl_alop_th_yn] = '1'	Were the therapy attempts effective?	dropdown, Required<br>1 Yes, completely<br>2 Yes, partially<br>0 No, not at all

145	cosm_probl_alop_th_act<br>Show the field ONLY if:<br>[cosm_probl_alop_th_yn] = '1'	Which of the therapy methods are you still currently implementing?	checkbox, Required<br>1 cosm_probl_alop_th_act___1 lifestyle intervention (diet, exercise, etc.)<br>2 cosm_probl_alop_th_act___2 medication (including contraceptive pill)<br>3 cosm_probl_alop_th_act___3 psychotherapy<br>4 cosm_probl_alop_th_act___4 bariatric surgery (e.g. gastric bypass)<br>88 cosm_probl_alop_th_act___88 other<br>0 cosm_probl_alop_th_act___0 no therapy anymore<br>Field Annotation: @NONEOFTHEABOVE=0
146	cosm_probl_alop_th1_act<br>Show the field ONLY if:<br>[cosm_probl_alop_th_act(1)] = '1'	Please select the lifestyle interventions you are still currently performing:	checkbox, Required<br>1 cosm_probl_alop_th1_act___1 Behavioural interventions (e.g. goal-setting, self-monitoring, slower eating, etc.)<br>2 cosm_probl_alop_th1_act___2 attitude<br>3 cosm_probl_alop_th1_act___3 dietary interventions (e.g. well-balanced, reduce energy intake, etc.)<br>4 cosm_probl_alop_th1_act___4 physical activity<br>5 cosm_probl_alop_th1_act___5 weight assessment and reduction<br>88 cosm_probl_alop_th1_act___88 other (as described above)
147	cosm_probl_alop_th2_act<br>Show the field ONLY if:<br>[cosm_probl_alop_th_act(2)] = '1'	Please select the medication you are still currently taking:	checkbox, Required<br>1 cosm_probl_alop_th2_act___1 contraceptive pill<br>2 cosm_probl_alop_th2_act___2 anti-androgens<br>3 cosm_probl_alop_th2_act___3 metformin<br>88 cosm_probl_alop_th2_act___88 other (as described above)
148	cosm_probl_alop_th_adv_yn<br>Show the field ONLY if:<br>[cosm_probl_alop_th_yn] = '1'	Have you been consulted by your gynecologist regarding the therapy attempts?	radio, Required<br>1 Yes<br>0 No
149	cosm_probl_alop_th_adv_sc<br>Show the field ONLY if:<br>[cosm_probl_alop_th_adv_yn] = '1'	Please rate your satisfaction with consultation regarding therapy attempts.<br>*Drag the blue slider and drop it at the desired position.*	slider (number), Required<br>Slider labels: 0, 50, 100<br>Custom alignment: RH
150	cosm_probl_alop_th_adv_wish<br>Show the field ONLY if:<br>[cosm_probl_alop_th_adv_yn] = '0'	Would you have wished for a consultation by your gynecologist regarding therapy attempts?	radio, Required<br>1 Yes<br>0 No

151	cosm_probl_hirs_info<br>Show the field ONLY if:<br>[clhy_hirsutism_yn] = '1'	Section Header: *hirsutism*<br>Earlier in this survey you stated to be affected by hirsutism. Please answer following questions about it.	descriptive
152	cosm_probl_hirs_bother_yn<br>Show the field ONLY if:<br>[cosm_probl(3)] = '1' or<br>[clhy_hirsutism_yn] = '1'	Does/did your hirsutism bother you?	radio, Required<br>1 Yes<br>0 No
153	cosm_probl_hirs_adv_yn<br>Show the field ONLY if:<br>[cosm_probl(3)] = '1' or<br>[clhy_hirsutism_yn] = '1'	Have you been consultet by your gynecologists about this?	radio, Required<br>1 Yes<br>0 No
154	cosm_probl_hirs_adv_sc<br>Show the field ONLY if:<br>[cosm_probl_hirs_adv_yn] = '1'	Please rate your satisfaction with consultation regarding hirsutism?<br>*Drag the blue slider and drop it at the desired position.*	slider (number), Required<br>Slider labels: 0, 50, 100<br>Custom alignment: RH
155	cosm_probl_hirs_adv_wish<br>Show the field ONLY if:<br>[cosm_probl_hirs_adv_yn] = '0'	Would you have wished for a consultation by your gynecologist regarding hirsutism?	radio, Required<br>1 Yes<br>0 No
156	cosm_probl_hirs_th_yn<br>Show the field ONLY if:<br>[cosm_probl(3)] = '1' or<br>[clhy_hirsutism_yn] = '1'	Have you tried any therapy for hirsutism?	radio, Required<br>1 Yes<br>0 No<br>99 Unknown
157	cosm_probl_hirs_th_spec<br>Show the field ONLY if:<br>[cosm_probl_hirs_th_yn] = '1'	Which therapy method have you tried already?<br>*If you are not sure about one of the options you can check it to have a look at the sub-categories. If there isn't anything suitable you can unckeck the option again.*	checkbox, Required<br>1 cosm_probl_hirs_th_spec___1 lifestyle intervention (diet, exercise, etc.)<br>2 cosm_probl_hirs_th_spec___2 medication (including contraceptive pill)<br>3 cosm_probl_hirs_th_spec___3 psychotherapy<br>4 cosm_probl_hirs_th_spec___4 bariatric surgery (e.g. gastric bypass)<br>88 cosm_probl_hirs_th_spec___88 other
158	cosm_probl_hirs_th_spec_88<br>Show the field ONLY if:<br>[cosm_probl_hirs_th_spec(88)] = '1'	What other therapeutic options?<br>*Please specify.*	notes, Required
159	cosm_probl_hirs_th1_spec<br>Show the field ONLY if:<br>[cosm_probl_hirs_th_spec(1)] = '1'	Which lifestyle interventions have you tried?	checkbox, Required<br>1 cosm_probl_hirs_th1_spec___1 Behavioural interventions (e.g. goal-setting, self-monitoring, slower eating, etc.)<br>2 cosm_probl_hirs_th1_spec___2 attitude<br>3 cosm_probl_hirs_th1_spec___3 dietary interventions (e.g. well-balanced, reduce energy intake, etc.)<br>4 cosm_probl_hirs_th1_spec___4 physical activity<br>5 cosm_probl_hirs_th1_spec___5 weight assessment and reduction<br>88 cosm_probl_hirs_th1_spec___88 other
160	cosm_probl_hirs_th1_spec_88<br>Show the field ONLY if:<br>[cosm_probl_hirs_th1_spec(88)] = '1'	What other lifestyle intervention?<br>*Please specify.*	notes, Required
161	cosm_probl_hirs_th2_spec<br>Show the field ONLY if:<br>[cosm_probl_hirs_th_spec(2)] = '1'	Please select the applicable medications:	checkbox, Required<br>1 cosm_probl_hirs_th2_spec___1 contraceptive pill<br>2 cosm_probl_hirs_th2_spec___2 anti-androgens<br>3 cosm_probl_hirs_th2_spec___3 metformin<br>88 cosm_probl_hirs_th2_spec___88 other
162	cosm_probl_hirs_th2_spec_88<br>Show the field ONLY if:<br>[cosm_probl_hirs_th2_spec(88)] = '1'	What other medication?<br>*Please specify.*	notes, Required
163	cosm_probl_hirs_th_effect<br>Show the field ONLY if:<br>[cosm_probl_hirs_th_yn] = '1'	Were the therapy attempts effective?	dropdown, Required<br>1 Yes, completely<br>2 Yes, partially<br>0 No, not at all
164	cosm_probl_hirs_th_act<br>Show the field ONLY if:<br>[cosm_probl_hirs_th_yn] = '1'	Which of the therapy methods are you still currently implementing?	checkbox, Required<br>1 cosm_probl_hirs_th_act___1 lifestyle intervention (diet, exercise, etc.)

			2 | cosm_probl_hirs_th_act 2 | medication (including contraceptive pill)<br>3 | cosm_probl_hirs_th_act 3 | psychotherapy<br>4 | cosm_probl_hirs_th_act 4 | bariatric surgery (e.g. gastric bypass)<br>88 | cosm_probl_hirs_th_act 88 | other<br>0 | cosm_probl_hirs_th_act 0 | no therapy anymore<br>Field Annotation: @NONEOFTHEABOVE=0
165	cosm_probl_hirs_th1_act<br>Show the field ONLY if:<br>[cosm_probl_hirs_th_act(1)] = '1'	Please select the lifestyle interventions you are still currently performing:	checkbox, Required<br>1 | cosm_probl_hirs_th1_act 1 | Behavioural interventions (e.g. goal-setting, self-monitoring, slower eating, etc.)<br>2 | cosm_probl_hirs_th1_act 2 | attitude<br>3 | cosm_probl_hirs_th1_act 3 | dietary interventions (e.g. well-balanced, reduce energy intake, etc.)<br>4 | cosm_probl_hirs_th1_act 4 | physical activity<br>5 | cosm_probl_hirs_th1_act 5 | weight assessment and reduction<br>88 | cosm_probl_hirs_th1_act 88 | other (as described above)
166	cosm_probl_hirs_th2_act<br>Show the field ONLY if:<br>[cosm_probl_hirs_th_act(2)] = '1'	Please select the medication you are still currently taking:	checkbox, Required<br>1 | cosm_probl_hirs_th2_act 1 | contraceptive pill<br>2 | cosm_probl_hirs_th2_act 2 | anti-androgens<br>3 | cosm_probl_hirs_th2_act 3 | metformin<br>88 | cosm_probl_hirs_th2_act 88 | other (as described above)
167	cosm_probl_hirs_th_adv_yn<br>Show the field ONLY if:<br>[cosm_probl_hirs_th_yn] = '1'	Have you been consulted by your gynecologist regarding the therapy attempts?	radio, Required<br>1 | Yes<br>0 | No
168	cosm_probl_hirs_th_adv_sc<br>Show the field ONLY if:<br>[cosm_probl_hirs_th_adv_yn] = '1'	Please rate your satisfaction with consultation regarding therapy attempts.<br>*Drag the blue slider and drop it at the desired position.*	slider (number), Required<br>Slider labels: 0, 50, 100<br>Custom alignment: RH
169	cosm_probl_hirs_th_adv_wish<br>Show the field ONLY if:<br>[cosm_probl_hirs_th_adv_yn] = '0'	Would you have wished for a consultation by your gynecologist regarding therapy attempts?	radio, Required<br>1 | Yes<br>0 | No

170	cosm_probl_adip_info1<br>Show the field ONLY if: [bmi] >= 25 and [bmi] < 30	Section Header: *overweight*<br>According to the calculation of your BMI, you suffer from slight overweight. Please answer the following questions about it.	descriptive
171	cosm_probl_adip_info2<br>Show the field ONLY if: [bmi] >= 30 and [bmi] < 40	According to the calculation of your BMI, you suffer from moderate overweight. Please answer the following questions about it.	descriptive
172	cosm_probl_adip_info3<br>Show the field ONLY if: [bmi] >= 40	According to the calculation of your BMI, you suffer from severe overweight. Please answer the following questions about it.	descriptive
173	cosm_probl_adip_bother_yn<br>Show the field ONLY if: [cosm_probl(4)] = '1' or [bmi] >= 25	Does/did your overweight bother you?	radio, Required<br>1 Yes<br>0 No
174	cosm_probl_adip_adv_yn<br>Show the field ONLY if: [cosm_probl(4)] = '1' or [bmi] >= 25	Have you been consultet by your gynecologists about this?	radio, Required<br>1 Yes<br>0 No
175	cosm_probl_adip_adv_sc<br>Show the field ONLY if: [cosm_probl_adip_adv_yn] = '1'	Please rate your satisfaction with consultation regarding overweight?<br>*Drag the blue slider and drop it at the desired position.*	slider (number), Required<br>Slider labels: 0, 50, 100<br>Custom alignment: RH
176	cosm_probl_adip_adv_wish<br>Show the field ONLY if: [cosm_probl_adip_adv_yn] = '0'	Would you have wished for a consultation by your gynecologist regarding overweight?	radio, Required<br>1 Yes<br>0 No
177	cosm_probl_adip_th_yn<br>Show the field ONLY if: [cosm_probl(4)] = '1' or [bmi] >= 25	Have you tried any therapy for overweight?	radio, Required<br>1 Yes<br>0 No<br>99 Unknown
178	cosm_probl_adip_th_spec<br>Show the field ONLY if: [cosm_probl_adip_th_yn] = '1'	Which therapy method have you tried already?<br>*If you are not sure about one of the options you can check it to have a look at the sub-categories. If there isn't anything suitable you can unckeck the option again.*	checkbox, Required<br>1 cosm_probl_adip_th_spec___1 lifestyle intervention (diet, exercise, etc.)<br>2 cosm_probl_adip_th_spec___2 medication (including contraceptive pill)<br>3 cosm_probl_adip_th_spec___3 psychotherapy<br>4 cosm_probl_adip_th_spec___4 bariatric surgery (e.g. gastric bypass)<br>88 cosm_probl_adip_th_spec___88 other
179	cosm_probl_adip_th_spec_88<br>Show the field ONLY if: [cosm_probl_adip_th_spec(88)] = '1'	What other therapeutic options?<br>*Please specify.*	notes, Required
180	cosm_probl_adip_th1_spec<br>Show the field ONLY if: [cosm_probl_adip_th_spec(1)] = '1'	Which lifestyle interventions have you tried?	checkbox, Required<br>1 cosm_probl_adip_th1_spec___1 Behavioural interventions (e.g. goal-setting, self-monitoring, slower eating, etc.)<br>2 cosm_probl_adip_th1_spec___2 attitude<br>3 cosm_probl_adip_th1_spec___3 dietary interventions (e.g. well-balanced, reduce energy intake, etc.)<br>4 cosm_probl_adip_th1_spec___4 physical activity<br>5 cosm_probl_adip_th1_spec___5 weight assessment and reduction<br>88 cosm_probl_adip_th1_spec___88 other
181	cosm_probl_adip_th1_spec_88<br>Show the field ONLY if: [cosm_probl_adip_th1_spec(88)] = '1'	What other lifestyle intervention?<br>*Please specify.*	notes, Required
182	cosm_probl_adip_th2_spec<br>Show the field ONLY if: [cosm_probl_adip_th_spec(2)] = '1'	Please select the applicable medications:	checkbox, Required<br>1 cosm_probl_adip_th2_spec___1 contraceptive pill<br>2 cosm_probl_adip_th2_spec___2 anti-androgens<br>3 cosm_probl_adip_th2_spec___3 metformin<br>88 cosm_probl_adip_th2_spec___88 other
183	cosm_probl_adip_th2_spec_88<br>Show the field ONLY if: [cosm_probl_adip_th2_spec(88)] = '1'	What other medication?<br>*Please specify.*	notes, Required
184	cosm_probl_adip_th_effect<br>Show the field ONLY if: [cosm_probl_adip_th_yn] = '1'	Were the therapy attempts effective?	dropdown, Required<br>1 Yes, completely

			2 Yes, partially<br>0 No, not at all
185	cosm_probl_adip_th_act<br>Show the field ONLY if:<br>[cosm_probl_adip_th_yn] = '1'	Which of the therapy methods are you still currently implementing?	checkbox, Required<br>1 cosm_probl_adip_th_act 1 lifestyle intervention (diet, exercise, etc.)<br>2 cosm_probl_adip_th_act 2 medication (including contraceptive pill)<br>3 cosm_probl_adip_th_act 3 psychotherapy<br>4 cosm_probl_adip_th_act 4 bariatric surgery (e.g. gastric bypass)<br>88 cosm_probl_adip_th_act 88 other<br>0 cosm_probl_adip_th_act 0 no therapy anymore<br>Field Annotation: @NONEOFTHEABOVE=0
186	cosm_probl_adip_th1_act<br>Show the field ONLY if:<br>[cosm_probl_adip_th_act(1)] = '1'	Please select the lifestyle interventions you are still currently performing:	checkbox, Required<br>1 cosm_probl_adip_th1_act 1 Behavioural interventions (e.g. goal-setting, self-monitoring, slower eating, etc.)<br>2 cosm_probl_adip_th1_act 2 attitude<br>3 cosm_probl_adip_th1_act 3 dietary interventions (e.g. well-balanced, reduce energy intake, etc.)<br>4 cosm_probl_adip_th1_act 4 physical activity<br>5 cosm_probl_adip_th1_act 5 weight assessment and reduction<br>88 cosm_probl_adip_th1_act 88 other (as described above)
187	cosm_probl_adip_th2_act<br>Show the field ONLY if:<br>[cosm_probl_adip_th_act(2)] = '1'	Please select the medication you are still currently taking:	checkbox, Required<br>1 cosm_probl_adip_th2_act 1 contraceptive pill<br>2 cosm_probl_adip_th2_act 2 anti-androgens<br>3 cosm_probl_adip_th2_act 3 metformin<br>88 cosm_probl_adip_th2_act 88 other (as described above)
188	cosm_probl_adip_th_adv_yn<br>Show the field ONLY if:<br>[cosm_probl_adip_th_yn] = '1'	Have you been consulted by your gynecologist regarding the therapy attempts?	radio, Required<br>1 Yes<br>0 No
189	cosm_probl_adip_th_adv_sc<br>Show the field ONLY if:<br>[cosm_probl_adip_th_adv_yn] = '1'	Please rate your satisfaction with consultation regarding therapy attempts.<br>*Drag the blue slider and drop it at the desired position.*	slider (number), Required<br>Slider labels: 0, 50, 100<br>Custom alignment: RH
190	cosm_probl_adip_th_adv_wish<br>Show the field ONLY if:<br>[cosm_probl_adip_th_adv_yn] = '0'	Would you have wished for a consultation by your gynecologist regarding therapy attempts?	radio, Required<br>1 Yes<br>0 No

191	cosm_probl_oth_bother_yn<br>Show the field ONLY if:<br>[cosm_probl(88)] = '1'	Section Header: *other*<br>You have selected to suffer from another cosmetic problem. You have described this problem in the text field above. Does/did this problem bother you?	radio, Required<br>1 Yes<br>0 No
192	cosm_probl_oth_adv_yn<br>Show the field ONLY if:<br>[cosm_probl(88)] = '1'	Have you been consultet by your gynecologists about this?	radio, Required<br>1 Yes<br>0 No
193	cosm_probl_oth_adv_sc<br>[cosm_probl_oth_adv_yn] = '1'	Please rate your satisfaction with consultation regarding your other problem?<br>*Drag the blue slider and drop it at the desired position.*	slider (number), Required<br>Slider labels: 0, 50, 100<br>Custom alignment: RH
194	cosm_probl_oth_adv_wish<br>Show the field ONLY if:<br>[cosm_probl_oth_adv_yn] = '0'	Would you have wished for a consultation by your gynecologist regarding your other problem?	radio, Required<br>1 Yes<br>0 No
195	cosm_probl_oth_th_yn<br>Show the field ONLY if:<br>[cosm_probl(88)] = '1'	Have you tried any therapy for your other problem (as described above)?	radio, Required<br>1 Yes<br>0 No<br>99 Unknown
196	cosm_probl_oth_th_spec<br>Show the field ONLY if:<br>[cosm_probl_oth_th_yn] = '1'	Which therapy method have you tried already?<br>*If you are not sure about one of the options you can check it to have a look at the sub-categories. If there isn't anything suitable you can unckeck the option again.*	checkbox, Required<br>1 cosm_probl_oth_th_spec 1 lifestyle intervention (diet, exercise, etc.)<br>2 cosm_probl_oth_th_spec 2 medication (including contraceptive pill)<br>3 cosm_probl_oth_th_spec 3 psychotherapy<br>4 cosm_probl_oth_th_spec 4 bariatric surgery (e.g. gastric bypass)<br>88 cosm_probl_oth_th_spec 88 other
197	cosm_probl_oth_th_spec_88<br>Show the field ONLY if:<br>[cosm_probl_oth_th_spec(88)] = '1'	What other therapeutic options?<br>*Please specify.*	notes, Required
198	cosm_probl_oth_th1_spec<br>Show the field ONLY if:<br>[cosm_probl_oth_th_spec(1)] = '1'	Which lifestyle interventions have you tried?	checkbox, Required<br>1 cosm_probl_oth_th1_spec 1 Behavioural interventions (e.g. goal-setting, self-monitoring, slower eating, etc.)<br>2 cosm_probl_oth_th1_spec 2 attitude<br>3 cosm_probl_oth_th1_spec 3 dietary interventions (e.g. well-balanced, reduce energy intake, etc.)<br>4 cosm_probl_oth_th1_spec 4 physical activity<br>5 cosm_probl_oth_th1_spec 5 weight assessment and reduction<br>88 cosm_probl_oth_th1_spec 88 other
199	cosm_probl_oth_th1_spec_88<br>Show the field ONLY if:<br>[cosm_probl_oth_th1_spec(88)] = '1'	What other lifestyle intervention?<br>*Please specify.*	notes, Required
200	cosm_probl_oth_th2_spec<br>Show the field ONLY if:<br>[cosm_probl_oth_th_spec(2)] = '1'	Please select the applicable medications:	checkbox, Required<br>1 cosm_probl_oth_th2_spec 1 contraceptive pill<br>2 cosm_probl_oth_th2_spec 2 anti-androgens<br>3 cosm_probl_oth_th2_spec 3 metformin<br>88 cosm_probl_oth_th2_spec 88 other
201	cosm_probl_oth_th2_spec_88<br>Show the field ONLY if:<br>[cosm_probl_oth_th2_spec(88)] = '1'	What other medication?<br>*Please specify.*	notes, Required
202	cosm_probl_oth_th_effect<br>Show the field ONLY if:<br>[cosm_probl_oth_th_yn] = '1'	Were the therapy attempts effective?	dropdown, Required<br>1 Yes, completely<br>2 Yes, partially<br>0 No, not at all
203	cosm_probl_oth_th_act<br>Show the field ONLY if:<br>[cosm_probl_oth_th_yn] = '1'	Which of the therapy methods are you still currently implementing?	checkbox, Required<br>1 cosm_probl_oth_th_act 1 lifestyle intervention (diet, exercise, etc.)<br>2 cosm_probl_oth_th_act 2 medication (including contraceptive pill)<br>3 cosm_probl_oth_th_act 3 psychotherapy<br>4 cosm_probl_oth_th_act 4 bariatric surgery (e.g. gastric bypass)

			88 cosm_probl_oth_th_act 88 other<br>0 cosm_probl_oth_th_act 0 none more<br>Field Annotation: @NONEOFTHEABOVE=0
204	cosm_probl_oth_th1_act<br>Show the field ONLY if:<br>[cosm_probl_oth_th_act(1)] = '1'	Please select the lifestyle interventions you are still currently performing:	checkbox, Required<br>1 cosm_probl_oth_th1_act 1 Behavioural interventions (e.g. goal-setting, self-monitoring, slower eating, etc.)<br>2 cosm_probl_oth_th1_act 2 attitude<br>3 cosm_probl_oth_th1_act 3 dietary interventions (e.g. well-balanced, reduce energy intake, etc.)<br>4 cosm_probl_oth_th1_act 4 physical activity<br>5 cosm_probl_oth_th1_act 5 weight assessment and reduction<br>88 cosm_probl_oth_th1_act 88 other (as described above)
205	cosm_probl_oth_th2_act<br>Show the field ONLY if:<br>[cosm_probl_oth_th_act(2)] = '1'	Please select the medication you are still currently taking:	checkbox, Required<br>1 cosm_probl_oth_th2_act 1 contraceptive pill<br>2 cosm_probl_oth_th2_act 2 anti-androgens<br>3 cosm_probl_oth_th2_act 3 metformin<br>88 cosm_probl_oth_th2_act 88 other (as described above)
206	cosm_probl_oth_th_adv_yn<br>Show the field ONLY if:<br>[cosm_probl_oth_th_yn] = '1'	Have you been consulted by your gynecologist regarding the therapy attempts?	radio, Required<br>1 Yes<br>0 No
207	cosm_probl_oth_th_adv_sc<br>Show the field ONLY if:<br>[cosm_probl_oth_th_adv_yn] = '1'	Please rate your satisfaction with consultation regarding therapy attempts.<br>*Drag the blue slider and drop it at the desired position.*	slider (number), Required<br>Slider labels: 0, 50, 100<br>Custom alignment: RH
208	cosm_probl_oth_th_adv_wish<br>Show the field ONLY if:<br>[cosm_probl_oth_th_adv_yn] = '0'	Would you have wished for a consultation by your gynecologist regarding therapy attempts?	radio, Required<br>1 Yes<br>0 No

209	cosm_satisf_sc	Section Header: *overall satisfaction*<br>Please rate your overall satisfaction with the medical care you receive from your gynecologist regarding cosmetic issues.<br>*Drag the blue slider and drop it at the desired position.*	slider (number), Required<br>Slider labels: 0, 50, 100<br>Custom alignment: RH
210	cosm_addit_yn	Would you like additional consultation?	radio, Required<br>1 Yes<br>0 No
211	cosm_addit_spec<br>Show the field ONLY if:<br>[cosm_addit_yn] = '1'	Please select what you would wish to have in addition:	checkbox, Required<br>1 cosm_addit_spec___1 More consultation and reassurance<br>2 cosm_addit_spec___2 More information providing (such as booklets)<br>3 cosm_addit_spec___3 More possibilities to ask questions<br>4 cosm_addit_spec___4 More examinations (blood tests, ultrasound, etc.)<br>5 cosm_addit_spec___5 More therapy options<br>88 cosm_addit_spec___88 Other
212	cosm_addit_spec_88<br>Show the field ONLY if:<br>[cosm_addit_spec(88)] = '1'	What other additional consultation?<br>*Please specify.*	notes, Required
213	einflussbereich_1_kosmetik_co mplete	Section Header: *Form Status*<br>Complete?	dropdown<br>0 Incomplete<br>1 Unverified<br>2 Complete

214	stw_disease	Do you have any of the following conditions? Please select the applicable ones.	checkbox, Required<br>1 stw_disease___1 Diabetes mellitus<br>2 stw_disease___2 Gestational diabetes (currently or in the past)<br>3 stw_disease___3 Prediabetes (elevated blood glucose but not yet diabetes)<br>0 stw_disease___0 None of the above<br>Field Annotation: @NONEOFTHEABOVE=0
215	stw_disease1_spec<br>Show the field ONLY if:<br>[stw_disease(1)] = '1'	What type of diabetes do you have?	radio, Required<br>1 Typ 1<br>2 Typ 2
216	stw_disease2_spec_yn<br>Show the field ONLY if:<br>[stw_disease(2)] = '1'	Did this diabetes disappear after pregnancy?	radio, Required<br>1 Yes<br>0 No
217	stw_screen_bz_yn	Have you ever had a blood glucose test done?<br>*This includes fasting blood glucose, long-term serum glucose (HbA1c) or oral glucose tolerance test (=oGTT, drink glucose solution followed by multiple measurements of glucose)*	radio, Required<br>1 Yes<br>0 No<br>99 Unknown
218	stw_disease_adv_yn<br>Show the field ONLY if:<br>[stw_disease(1)] = '1' or [stw_disease(2)] = '1' or [stw_disease(3)] = '1'	Have you been consultet by your gynecologists about your diabetic disorder?	radio, Required<br>1 Yes<br>0 No
219	stw_disease_adv_sc<br>Show the field ONLY if:<br>[stw_disease_adv_yn] = '1'	Please rate your satisfaction with consultation regarding diabetic disorders?<br>*Drag the blue slider and drop it at the desired position.*	slider (number), Required<br>Slider labels: 0, 50, 100<br>Custom alignment: RH
220	stw_disease_adv_hadb_yn<br>Show the field ONLY if:<br>[stw_disease(1)] = '1' or [stw_disease(2)] = '1' or [stw_disease(3)] = '1'	Have you been consultet by your primary care physician or diabetologist about your diabetic disorder?	radio, Required<br>1 Yes<br>0 No
221	stw_disease_adv_wish<br>Show the field ONLY if:<br>[stw_disease_adv_yn] = '0'	Would you have wished for a consultation by your gynecologist regarding diabetic disorders?	radio, Required<br>1 Yes<br>0 No
222	stw_diseases_th_yn<br>Show the field ONLY if:<br>[stw_disease(1)] = '1' or [stw_disease(2)] = '1' or [stw_disease(3)] = '1'	Section Header: *therapy diabetic disorder*<br>Have you tried any therapy for diabetic disorders?	radio, Required<br>1 Yes<br>0 No<br>99 Unknown
223	stw_diseases_th_spec<br>Show the field ONLY if:<br>[stw_diseases_th_yn] = '1'	Which therapy method have you tried already?<br>*If you are not sure about one of the options you can select it to have a look at the sub-categories. If there isn't anything suitable you can unckeck the option again.*	checkbox, Required<br>1 stw_diseases_th_spec___1 lifestyle intervention (diet, exercise, etc.)<br>2 stw_diseases_th_spec___2 medication (including contraceptive pill)<br>3 stw_diseases_th_spec___3 psychotherapy<br>4 stw_diseases_th_spec___4 bariatric surgery (e.g. gastric bypass)<br>88 stw_diseases_th_spec___88 other
224	stw_diseases_th_spect_88<br>Show the field ONLY if:<br>[stw_diseases_th_spec(88)] = '1'	What other therapeutic options?<br>*Please specify.*	notes, Required
225	stw_disease_th1_spec<br>Show the field ONLY if:<br>[stw_diseases_th_spec(1)] = '1'	Which lifestyle interventions have you tried?	checkbox, Required<br>1 stw_disease_th1_spec___1 Behavioural interventions (e.g. goal-setting, self-monitoring, slower eating, etc.)<br>2 stw_disease_th1_spec___2 attitude<br>3 stw_disease_th1_spec___3 dietary interventions (e.g. well-balanced, reduce energy intake, etc.)<br>4 stw_disease_th1_spec___4 physical activity<br>5 stw_disease_th1_spec___5 weight assessment and reduction<br>88 stw_disease_th1_spec___88 other (as described above)
226	stw_disease_th1_spec_88<br>Show the field ONLY if:<br>[stw_disease_th1_spec(88)] = '1'	What other lifestyle intervention?<br>*Please specify.*	notes, Required

227	stw_disease_th2_spec<br>Show the field ONLY if:<br>[stw_diseases_th_spec(2)] = '1'	Please select the applicable medications:	checkbox, Required<br>1 stw_disease_th2_spec___1 Metformin<br>2 stw_disease_th2_spec___2 other oral antidiabetics (pills)<br>3 stw_disease_th2_spec___3 Insulin (injection/pen)<br>88 stw_disease_th2_spec___88 other
228	stw_disease_th2_spec_88<br>Show the field ONLY if:<br>[stw_disease_th2_spec(88)] = '1'	What other medication?<br>*Please specify.*	notes, Required
229	stw_disease_th_effect<br>Show the field ONLY if:<br>[stw_diseases_th_yn] = '1'	Were the therapy attempts effective?	dropdown, Required<br>1 Yes, completely<br>2 Yes, partially<br>0 No, not at all
230	stw_diseases_th_act<br>Show the field ONLY if:<br>[stw_diseases_th_yn] = '1'	Which of the therapy methods are you still currently implementing?	checkbox, Required<br>1 stw_diseases_th_act___1 lifestyle intervention (diet, exercise, etc.)<br>2 stw_diseases_th_act___2 medication (including contraceptive pill)<br>3 stw_diseases_th_act___3 psychotherapy<br>4 stw_diseases_th_act___4 bariatric surgery (e.g. gastric bypass)<br>88 stw_diseases_th_act___88 other<br>0 stw_diseases_th_act___0 Keine mehr
231	stw_disease_th1_act<br>Show the field ONLY if:<br>[stw_diseases_th_act(1)] = '1'	Please select the lifestyle interventions you are still currently performing:	checkbox, Required<br>1 stw_disease_th1_act___1 Behavioural interventions (e.g. goal-setting, self-monitoring, slower eating, etc.)<br>2 stw_disease_th1_act___2 attitude<br>3 stw_disease_th1_act___3 dietary interventions (e.g. well-balanced, reduce energy intake, etc.)<br>4 stw_disease_th1_act___4 physical activity<br>5 stw_disease_th1_act___5 weight assessment and reduction<br>88 stw_disease_th1_act___88 other (as described above)
232	stw_disease_th2_act<br>Show the field ONLY if:<br>[stw_diseases_th_act(2)] = '1'	Please select the medication you are still currently taking:	checkbox, Required<br>1 stw_disease_th2_act___1 Metformin<br>2 stw_disease_th2_act___2 other oral antidiabetics (pills)<br>3 stw_disease_th2_act___3 Insulin (injection/pen)<br>88 stw_disease_th2_act___88 other
233	stw_disease_th_adv_yn<br>Show the field ONLY if:<br>[stw_diseases_th_yn] = '1'	Have you been consulted by your gynecologist regarding the therapy attempts?	radio, Required<br>1 Yes<br>0 No
234	stw_disease_th_adv_sc<br>Show the field ONLY if:<br>[stw_disease_th_adv_yn] = '1'	Please rate your satisfaction with consultation regarding therapy attempts.<br>*Drag the blue slider and drop it at the desired position.*	slider (number), Required<br>Slider labels: 0, 50, 100<br>Custom alignment: RH
235	stw_disease_th_adv_wish<br>Show the field ONLY if:<br>[stw_disease_th_adv_yn] = '0'	Would you have wished for a consultation by your gynecologist regarding therapy attempts?	radio, Required<br>1 Yes<br>0 No
236	stw_satisf_sc<br>Show the field ONLY if:<br>[stw_disease(1)] = '1' or [stw_disease(2)] = '1' or [stw_disease(3)] = '1'	Section Header: *overall satisfaction metabolism / diabetic disorders*<br>Please rate your overall satisfaction with the medical care you receive from your gynecologist regarding diabetic disorders?<br>*Drag the blue slider and drop it at the desired position.*	slider (number), Required<br>Slider labels: 0, 50, 100<br>Custom alignment: RH
237	stw_addit_yn<br>Show the field ONLY if:<br>[stw_disease(1)] = '1' or [stw_disease(2)] = '1' or [stw_disease(3)] [illegible]	Would you like additional consultation?	radio, Required<br>1 Yes<br>0 No
238	stw_addit_spec<br>Show the field ONLY if:<br>[stw_addit_yn] = '1'	Please select what you would wish to have in addition:	checkbox, Required<br>1 stw_addit_spec___1 More consultation and reassurance<br>2 stw_addit_spec___2 More information providing (such as booklets)

			3 stw_addit_spec___3 More possibilities to ask questions<br>4 stw_addit_spec___4 More examinations (blood tests, ultrasound, etc.)<br>5 stw_addit_spec___5 More therapy options<br>88 stw_addit_spec___88 Other
239	stw_addit_spec_88<br>Show the field ONLY if:<br>[stw_addit_spec(88)] = '1'	What other additional consultation?<br>*Please specify.*	notes, Required
240	einflussbereich_2_stoffwechsel_complete	Section Header: *Form Status*<br>Complete?	dropdown<br>0 Incomplete<br>1 Unverified<br>2 Complete

241	endo_current_bother_yn<br>Show the field ONLY if:<br>[mens_current_regular_yn] = '0'	Section Header: *currently irregular menstruation*<br>Earlier in this survey you stated to currently have an irregular menstrual cycle. Do these irregularities bother you?	radio, Required<br>1 Yes<br>0 No
242	endo_current_adv_yn<br>Show the field ONLY if:<br>[mens_current_regular_yn] = '0'	Have you been consultet by your gynecologists about your currently irregular menstruation?	radio, Required<br>1 Yes<br>0 No
243	endo_current_adv_sc<br>Show the field ONLY if:<br>[endo_current_adv_yn] = '1'	Please rate your satisfaction with consultation regarding your currently irregular menstruation?<br>*Drag the blue slider and drop it at the desired position.*	slider (number), Required<br>Slider labels: 0, 50, 100<br>Custom alignment: RH
244	endo_current_adv_wish<br>Show the field ONLY if:<br>[endo_current_adv_yn] = '0'	Would you have wished for a consultation by your gynecologist regarding your currently irregular menstruation?	radio, Required<br>1 Yes<br>0 No
245	endo_past_bother_yn<br>Show the field ONLY if:<br>[mens_past_regular_yn] = '0'	Section Header: *past - irregular menstrual cycle profile*<br>Earlier in this survey you stated to have had an irregular menstrual cycle in the past. Did these irregularities bother you?	radio, Required<br>1 Yes<br>0 No
246	endo_past_adv_yn<br>Show the field ONLY if:<br>[mens_past_regular_yn] = '0'	Have you been consultet by your gynecologists about your irregular menstruation at that time?	radio, Required<br>1 Yes<br>0 No
247	endo_past_adv_sc<br>Show the field ONLY if:<br>[endo_past_adv_yn] = '1'	Please rate your satisfaction with consultation regarding your irregular menstruation at that time?<br>*Drag the blue slider and drop it at the desired position.*	slider (number), Required<br>Slider labels: 0, 50, 100<br>Custom alignment: RH
248	endo_past_adv_wish<br>Show the field ONLY if:<br>[endo_past_adv_yn] = '0'	Would you have wished for a consultation by your gynecologist regarding irregular menstruation at that time?	radio, Required<br>1 Yes<br>0 No
249	endo_pill_adv_yn<br>Show the field ONLY if:<br>[mens_current_regular_yn] = '0' or [mens_past_regular_yn] = '0'	Section Header: *contraceptive pill*<br>Did your gynecologist consult you about the option of using a contraceptive pill to treat irregular menstruations?<br>*This is only about whether the contraceptive pill was ever discussed. This is independent of whether it was desired or taken afterwards.*	radio, Required<br>1 Yes<br>0 No
250	endo_pill_adv_take_yn<br>Show the field ONLY if:<br>[endo_pill_adv_yn] = '1'	Following that, have you taken the contraceptive pill?	radio, Required<br>1 Yes<br>0 No
251	endo_pill_nadv_take_yn<br>Show the field ONLY if:<br>[endo_pill_adv_yn] = '0' or [endo_pill_adv_yn] = '1'	Have you taken the contraceptive pill (also) because of other reasons than irregular menstruations?	radio, Required<br>1 Yes<br>0 No
252	endo_pill_nadv_take_spec<br>Show the field ONLY if:<br>[endo_pill_nadv_take_yn] = '1'	For what reasons?	checkbox, Required<br>1 endo_pill_nadv_take_spec___1 acne<br>2 endo_pill_nadv_take_spec___2 alopecia<br>3 endo_pill_nadv_take_spec___3 excess hair growth<br>4 endo_pill_nadv_take_spec___4 contraception<br>88 endo_pill_nadv_take_spec___88 other
253	endo_pill_nadv_take_spec_88<br>Show the field ONLY if:<br>[endo_pill_nadv_take_spec(88)] = '1'	What other reasons?<br>*Please specify.*	notes, Required
254	endo_pill_effect<br>Show the field ONLY if:<br>[endo_pill_adv_take_yn] = '1' and [endo_pill_nadv_take_yn] = '1'	Was the pill effective against irregular menstruation?	dropdown, Required<br>1 Yes, completely<br>2 Yes, partially<br>0 No, not at all
255	endo_pill_effect_88<br>Show the field ONLY if:<br>[endo_pill_adv_take_yn] = '1' and [endo_pill_nadv_take_yn] = '1'	Was the pill effective against other mentionned problems?	dropdown, Required<br>1 Yes, completely<br>2 Yes, partially<br>0 No, not at all
256	endo_pill_take_act<br>Show the field ONLY if:<br>[endo_pill_adv_take_yn] = '1' or [endo_pill_nadv_take_yn] = '1'	Are you currently still taking the contraceptive pill?	radio, Required<br>1 Yes<br>0 No

257	endo_pill_stop_spec<br>Show the field ONLY if:<br>[endo_pill_take_act] = '0'	Why did you stop taking the contraceptive pill?	checkbox, Required<br>1 endo_pill_stop_spec___1 It helped too little against the problems<br>2 endo_pill_stop_spec___2 I do no longer want to take hormones<br>3 endo_pill_stop_spec___3 desire to have children<br>4 endo_pill_stop_spec___4 side effects<br>88 endo_pill_stop_spec___88 other
258	endo_pill_stop_spec_88<br>Show the field ONLY if:<br>[endo_pill_stop_spec(88)] = '1'	What other reasons?<br>*Please specify.*	notes, Required
259	endo_pill_ntake_spec<br>Show the field ONLY if:<br>[endo_pill_adv_take_yn] = '0'	Why did you decide not to take the pill?	checkbox, Required<br>2 endo_pill_ntake_spec___2 I don't want to take hormones<br>3 endo_pill_ntake_spec___3 desire to have children<br>4 endo_pill_ntake_spec___4 fear of side effects<br>88 endo_pill_ntake_spec___88 other
260	endo_pill_ntake_spec_88<br>Show the field ONLY if:<br>[endo_pill_nadv_take_spec(88)] = '1'	What other reasons?<br>*Please specify.*	notes, Required
261	endo_pill_adv_sc<br>Show the field ONLY if:<br>[endo_pill_adv_yn] = '1'	Please rate your satisfaction with consultation regarding contraceptive pill?<br>*Drag the blue slider and drop it at the desired position.*	slider (number), Required<br>Slider labels: 0, 50, 100<br>Custom alignment: RH
262	endo_pill_adv_wish<br>Show the field ONLY if:<br>[endo_pill_adv_yn] = '0'	Would you have wished for a consultation by your gynecologist regarding contraceptive pill?	radio, Required<br>1 Yes<br>0 No
263	endo_satisf_sc	Section Header: *overall satisfaction endometrum / menstrual cycle profile*<br>Please rate your overall satisfaction with the medical care you receive from your gynecologist regarding menstural cycle profile?<br>*Drag the blue slider and drop it at the desired position.*	slider (number), Required<br>Slider labels: 0, 50, 100<br>Custom alignment: RH
264	endo_addit_yn	Would you like additional consultation?	radio, Required<br>1 Yes<br>0 No
265	endo_addit_spec<br>Show the field ONLY if:<br>[endo_addit_yn] = '1'	Please select what you would wish to have in addition:	checkbox, Required<br>1 endo_addit_spec___1 More consultation and reassurance<br>2 endo_addit_spec___2 More information providing (such as booklets)<br>3 endo_addit_spec___3 More possibilities to ask questions<br>4 endo_addit_spec___4 More examinations (blood tests, ultrasound, etc.)<br>5 endo_addit_spec___5 More therapy options<br>88 endo_addit_spec___88 Other
266	endo_addit_spec_88<br>Show the field ONLY if:<br>[endo_addit_spec(88)] = '1'	What other additional consultation?<br>*Please specify.*	notes, Required
267	einflussbereich_3_endometrium_complete	Section Header: *Form Status*<br>Complete?	dropdown<br>0 Incomplete<br>1 Unverified<br>2 Complete

268	kw_yn	Section Header: *fertility*<br>Do you ever wish to have children?	radio, Required<br>1 Yes<br>0 No<br>99 Unknown
269	kw_act_yn<br>Show the field ONLY if: [kw_yn] = '1'	Do you currently want children?<br>*This refers to te wish to have children as soon as possible. For this, no attempts to become pregnant must have taken place yet.*	radio, Required<br>1 Yes<br>0 No
270	kw_act_nr<br>Show the field ONLY if: [kw_act_yn] = '1'	Approximately how long have you already been wanting to have children?	dropdown, Required<br>1 up to 1 month<br>2 1-3 months<br>3 3-6 months<br>4 6-9 months<br>5 9-12 months<br>6 1-2 years<br>7 2-3 years<br>8 3-4 years<br>9 more than 4 years<br>99 Unknown
271	kw_nact_nr<br>Show the field ONLY if: [kw_act_yn] = '0'	In appoximateley how many years do you wish to have children?	dropdown, Required<br>1 1<br>2 2<br>3 3<br>4 4<br>5 5<br>6 6<br>7 7<br>8 8<br>9 9<br>10 10<br>11 über 10<br>99 Unknown<br>Custom alignment: RH
272	kw_adv_yn<br>Show the field ONLY if: [kw_yn] = '1' or [kw_yn] = '99'	Have you been consultet by your gynecologists about fertility?<br>*Informed about possible difficulties and possibilities.*	radio, Required<br>1 Yes<br>0 No
273	kw_adv_sc<br>Show the field ONLY if: [kw_adv_yn] = '1'	Please rate your satisfaction with consultation regarding fertility?<br>*Drag the blue slider and drop it at the desired position.*	slider (number), Required<br>Slider labels: 0, 50, 100<br>Custom alignment: RH
274	kw_adv_wish<br>Show the field ONLY if: [kw_adv_yn] = '0'	Would you have wished for a consultation by your gynecologist regarding fertility?	radio, Required<br>1 Yes<br>0 No
275	kw_ss_yn<br>Show the field ONLY if: [children_nr] = '0'	Section Header: *pregnancy*<br>Have you ever been pregnant?	radio, Required<br>1 Yes<br>0 No
276	kw_ss_nr<br>Show the field ONLY if: [kw_ss_yn] = '1' or ([children_nr] = '1' or [children_nr] = '2' or [chil dren_nr] = '3' or [children_nr] = '4' or [children_nr] = '5' or [childr en_nr] = '6' or [children_nr] = '7' or [children_nr] = '8' or [children _nr] = '9' or [children_nr] = '10' o r [children_nr] = '11')	How many times have you been pregnant?<br>*Including miscarriages and/or elective abortions. Please write the number of pregnancies (e.g. 3).*	text (number, Min: 1), Required
277	kw_ss_abort_fehl_yn<br>Show the field ONLY if: [kw_ss_yn] = '1'	Have you ever had a miscarriage?	radio, Required<br>1 Yes<br>0 No
278	kw_ss_abort_fehl_nr<br>Show the field ONLY if: [kw_ss_abort_fehl_yn] = '1'	How many pregnancies ended in miscarriage??<br>*Please write a number.*	text (number, Min: 1), Required

279	kw_ss_abort_fehl_time<br>Show the field ONLY if:<br>[kw_ss_abort_fehl_yn] = '1'	How far advanced was the pregnancy when you suffered a miscarriage.<br>*If you have had multiple miscarriages, you can select multiple fields..*	checkbox, Required<br>1 | kw_ss_abort_fehl_time___1 | < 12th week of pregnancy (early miscarriage)<br>2 | kw_ss_abort_fehl_time___2 | 12th - 22th week of pregnancy (late miscarriage)<br>3 | kw_ss_abort_fehl_time___3 | >22th week of pregnancy and / or weight >500g (stillbirth)
280	kw_ss_abort_abbr_yn<br>Show the field ONLY if: [kw_ss_yn] = '1'	Have you ever had an elective abortion?	radio, Required<br>1 | Yes<br>0 | No
281	kw_ss_abort_abbr_nr<br>Show the field ONLY if:<br>[kw_ss_abort_abbr_yn] = '1'	How many pregnancies ended in an elective abortion?<br>*Please write a number.*	text (number, Min: 1), Required
282	kw_ss_infert_yn	Have you ever tried to get pregnant for more than 1 year without success?<br>*Trying to get pregnant = regular, unprotected sexual intercourse at the right time of the menstrual cycle*	radio, Required<br>1 | Yes<br>0 | No
283	kw_ss_infert_act_yn<br>Show the field ONLY if:<br>[kw_ss_infert_yn] = '1'	Are you currently trying to get pregnant for more than a year?	radio, Required<br>1 | Yes<br>0 | No
284	kw_ss_infert_act_nr<br>Show the field ONLY if:<br>[kw_ss_infert_act_yn] = '1'	How long have you already been trying to get pregnant?	dropdown, Required<br>1 | 12 months<br>2 | 12-18 months<br>3 | 18-24 months<br>4 | 24-36 months<br>5 | 36-48 months<br>6 | more than 4 years
285	kw_ss_infert_past_nr<br>Show the field ONLY if:<br>[kw_ss_infert_act_yn] = '0'	How long has it been since you unsuccessfully tried to get pregnant for more than a year?<br>*Please give the number in full years (z.B.: 4)*	text (number), Required
286	kw_ss_infert_past_trynr<br>Show the field ONLY if:<br>[kw_ss_infert_act_yn] = '0'	For how long did you unsuccessfully try to get pregnant at that time?	dropdown, Required<br>1 | 12 months<br>2 | 12-18 months<br>3 | 18-24 months<br>4 | 24-36 months<br>5 | 36-48 months<br>6 | more than 4 years
287	kw_ss_infert_adv_yn<br>Show the field ONLY if:<br>[kw_ss_infert_yn] = '1'	Have you been consultet by your gynecologists about these unsuccessful pregnancy attempts?<br>*Informed about possible reasons and possibilities.*	radio, Required<br>1 | Yes<br>0 | No
288	kw_ss_infert_adv_sc<br>Show the field ONLY if:<br>[kw_ss_infert_adv_yn] = '1'	Please rate your satisfaction with consultation regarding unsuccessful pregnancy attempts?<br>*Drag the blue slider and drop it at the desired position.*	slider (number), Required<br>Slider labels: 0, 50, 100<br>Custom alignment: RH
289	kw_ss_infert_adv_wish<br>Show the field ONLY if:<br>[kw_ss_infert_adv_yn] = '0'	Would you have wished for a consultation by your gynecologist regarding unsuccessful pregnancy attempts?	radio, Required<br>1 | Yes<br>0 | No
290	kw_ss_infert_th_yn<br>Show the field ONLY if:<br>[kw_ss_infert_yn] = '1'	Have you tried any therapy to get pregnant?<br>*If you are not sure about that you can select "Yes" to have a look at the therapy options. If there isn't anything suitable you can change to "No" again.*	1 | Yes<br>0 | No
291	kw_ss_infert_th_spec<br>Show the field ONLY if:<br>[kw_ss_infert_th_yn] = '1'	Which therapy method have you tried already?<br>*If you are not sure about one of the options you can select it to have a look at the sub-categories. If there isn't anything suitable you can unckeck the option again.*	checkbox, Required<br>1 | kw_ss_infert_th_spec___1 | lifestyle intervention (diet, exercise, etc.)<br>2 | kw_ss_infert_th_spec___2 | medication (pills or injections for stimulation of egg maturation)<br>3 | kw_ss_infert_th_spec___3 | surgery<br>4 | kw_ss_infert_th_spec___4 | in-vitro fertilization (fertilized in the laboratory)<br>88 | kw_ss_infert_th_spec___88 | other
292	kw_ss_infert_th_spec_88<br>Show the field ONLY if:<br>[kw_ss_infert_th_spec(88)] = '1'	What other therapeutic options?<br>*Please specify.*	notes, Required

293	kw_ss_infert_th1_spec<br>Show the field ONLY if:<br>[kw_ss_infert_th_spec(1)] = '1'	Which lifestyle interventions have you tried?	checkbox, Required<br>1 | kw_ss_infert_th1_spec___1 | behavioural interventions (e.g. goal-setting, self-monitoring, slower eating, etc.)<br>2 | kw_ss_infert_th1_spec___2 | attitude<br>3 | kw_ss_infert_th1_spec___3 | dietary interventions (e.g. well-balanced, reduce energy intake, etc.)<br>4 | kw_ss_infert_th1_spec___4 | physical activity<br>5 | kw_ss_infert_th1_spec___5 | weight assessment and reduction<br>6 | kw_ss_infert_th1_spec___6 | blood glucose regulation<br>7 | kw_ss_infert_th1_spec___7 | blood pressure regulation<br>8 | kw_ss_infert_th1_spec___8 | abstinence from smoking, alcohol and other drugs<br>9 | kw_ss_infert_th1_spec___9 | sleep regulation<br>10 | kw_ss_infert_th1_spec___10 | changes regarding sexual life<br>88 | kw_ss_infert_th1_spec___88 | other
294	kw_ss_infert_th1_spec_88<br>Show the field ONLY if:<br>[kw_ss_infert_th1_spec(88)] = '1'	What other lifestyle intervention?<br>*Please specify.*	notes, Required
295	kw_ss_infert_th2_spec<br>Show the field ONLY if:<br>[kw_ss_infert_th_spec(2)] = '1'	Please select the applicable medications::<br>*If you do not remember what medications you received, select "Unknown".*	checkbox, Required<br>1 | kw_ss_infert_th2_spec___1 | Letrozole (pills, ="Femara")<br>2 | kw_ss_infert_th2_spec___2 | Clomiphene (pills, ="Serophene" oder "Clomid")<br>3 | kw_ss_infert_th2_spec___3 | Metformin (pills)<br>4 | kw_ss_infert_th2_spec___4 | Gonadotropines (injections, e.g. "Choriomon", "Menopur", "Merional", "Fostimon", "Gonal-F", etc.)<br>88 | kw_ss_infert_th2_spec___88 | other<br>99 | kw_ss_infert_th2_spec___99 | Unknown<br>Field Annotation: @NONEOFTHEABOVE=99
296	kw_ss_infert_th2_spec_88<br>Show the field ONLY if:<br>[kw_ss_infert_th2_spec(88)] = '1'	What other medication?<br>*Please specify.*	notes, Required
297	kw_ss_infert_th3_spec<br>Show the field ONLY if:<br>[kw_ss_infert_th_spec(3)] = '1'	Which operations were performed?<br>*If you do not remember what was operated, select "Unknown".*	checkbox, Required<br>1 | kw_ss_infert_th3_spec___1 | surgery on ovaries<br>2 | kw_ss_infert_th3_spec___2 | tubal patency testing (=chromopertubation/blue dye testing)<br>3 | kw_ss_infert_th3_spec___3 | bariatric surgery (e.g. gastric bypass)<br>88 | kw_ss_infert_th3_spec___88 | other<br>99 | kw_ss_infert_th3_spec___99 | Unknown<br>Field Annotation: @NONEOFTHEABOVE=99
298	kw_ss_infert_th3_spec_88<br>Show the field ONLY if:<br>[kw_ss_infert_th3_spec(88)] = '1'	What other operation?<br>*Please specify.*	notes, Required
299	kw_ss_infert_th_effect_ss_yn<br>Show the field ONLY if: [kw_ss_yn] = '1' and [kw_ss_infer t_th_yn] = '1'	Did you become pregnant with the help of any of these therapies?	radio, Required<br>1 | Yes<br>0 | No
300	kw_ss_infert_th_effect_kid_yn<br>Show the field ONLY if:<br>[kw_ss_infert_th_yn] = '1' and ([c hildren_nr] = '1' or [children_nr] = '2' or [children_nr] = '3' or [chil dren_nr] = '4' or [children_nr] = '5' or [children_nr] = '6' or [childr en_nr] = '7' or [children_nr] = '8' or [children_nr] = '9' or [children _nr] = '10' or [children_nr] = '11')	Do you have children conceived with the help of such therapy?	radio, Required<br>1 | Yes<br>0 | No

301	kw_ss_infert_th_effect_kid_nr<br>Show the field ONLY if:<br>[kw_ss_infert_th_effect_kid_yn] = '1' and ([children_nr] = '2' or [chil dren_nr] = '3' or [children_nr] = '4' or [children_nr] = '5' or [childr en_nr] = '6' or [children_nr] = '7' or [children_nr] = '8' or [children _nr] = '9' or [children_nr] = '10' o r [children_nr] = '11')	How many of your children have been conceived with the help of such therapy?	dropdown, Required<br>100 Alle<br>1 1<br>2 2<br>3 3<br>4 4<br>5 5<br>6 6<br>7 7<br>8 8<br>9 9<br>10 10<br>11 über 10
302	kw_ss_infert_th_act_yn<br>Show the field ONLY if:<br>[kw_ss_infert_th_yn] = '1'	Are you currently implementing any of the therapy methods or do you already have any of them planned?	radio, Required<br>1 Yes<br>0 No
303	kw_ss_infert_th_act_spec<br>Show the field ONLY if:<br>[kw_ss_infert_th_act_yn] = '1'	Which of the therapy methods are you still currently implementing or do you have already planned?	checkbox, Required<br>1 kw_ss_infert_th_act_spec_1 lifestyle intervention (diet, exercise, etc.)<br>2 kw_ss_infert_th_act_spec_2 medication (pills or injections for stimulation of egg maturation)<br>3 kw_ss_infert_th_act_spec_3 surgery<br>4 kw_ss_infert_th_act_spec_4 in-vitro fertilization (fertilized in the laboratory)<br>88 kw_ss_infert_th_act_spec_88 other
304	kw_ss_infert_th1_act_spec<br>Show the field ONLY if:<br>[kw_ss_infert_th_act_spec(1)] ='1'	Please select the lifestyle interventions you are still currently performing:	checkbox, Required<br>1 kw_ss_infert_th1_act_spec_1 behavioural interventions (e.g. goal-setting, self-monitoring, slower eating, etc.)<br>2 kw_ss_infert_th1_act_spec_2 attitude<br>3 kw_ss_infert_th1_act_spec_3 dietary interventions (e.g. well-balanced, reduce energy intake, etc.)<br>4 kw_ss_infert_th1_act_spec_4 physical activity<br>5 kw_ss_infert_th1_act_spec_5 weight assessment and reduction<br>6 kw_ss_infert_th1_act_spec_6 blood glucose regulation<br>7 kw_ss_infert_th1_act_spec_7 blood pressure regulation<br>8 kw_ss_infert_th1_act_spec_8 abstinence from smoking, alcohol and other drugs<br>9 kw_ss_infert_th1_act_spec_9 sleep regulation<br>10 kw_ss_infert_th1_act_spec_10 changes regarding sexual life<br>88 kw_ss_infert_th1_act_spec_88 other
305	kw_ss_infert_th2_act_spec<br>Show the field ONLY if:<br>[kw_ss_infert_th_act_spec(2)] ='1'	Please select the medication you are still currently taking::<br>*If you do not remember what medications you receive, select "Unknown".*	checkbox, Required<br>1 kw_ss_infert_th2_spec___1 Letrozole (pills, ="Femara")<br>2 kw_ss_infert_th2_spec___2 Clomiphene (pills, ="Serophene" oder "Clomid")<br>3 kw_ss_infert_th2_spec___3 Metformin (pills)<br>4 kw_ss_infert_th2_spec___4 Gonadotropines (injections, e.g. "Choriomon", "Menopur", "Merional", "Fostimon", "Gonal-F", etc.)<br>88 kw_ss_infert_th2_spec___88 other<br>99 kw_ss_infert_th2_spec___99 Unknown<br>Field Annotation: @NONEOFTHEABOVE=99
306	kw_ss_infert_th3_act_spec<br>Show the field ONLY if:<br>[kw_ss_infert_th_act_spec(3)] ='1'	Please select the operations that are currently planned:<br>*If you do not remember what will be operated, select "Unknown".*	checkbox, Required<br>1 kw_ss_infert_th3_spec___1 surgery on ovaries<br>2 kw_ss_infert_th3_spec___2 tubal patency testing (=chromopertubation/blue dye testing)

			3 kw_ss_infert_th3_spec___3 bariatric surgery (e.g. gastric bypass)<br>88 kw_ss_infert_th3_spec___88 other<br>99 kw_ss_infert_th3_spec___99 Unknown<br>Field Annotation: @NONEOFTHEABOVE=99
307	kw_ss_infert_th_adv_yn<br>Show the field ONLY if:<br>[kw_ss_infert_th_yn] = '1'	Have you been consulted by your gynecologist regarding the therapy attempts?	radio, Required<br>1 Yes<br>0 No
308	kw_ss_infert_th_adv_sc<br>Show the field ONLY if:<br>[kw_ss_infert_th_adv_yn] = '1'	Please rate your satisfaction with consultation regarding therapy attempts.<br>*Drag the blue slider and drop it at the desired position.*	slider (number), Required<br>Slider labels: 0, 50, 100<br>Custom alignment: RH
309	kw_ss_infert_th_adv_wish<br>Show the field ONLY if:<br>[kw_ss_infert_th_adv_yn] = '0'	Would you have wished for a consultation by your gynecologist regarding therapy attempts?	radio, Required<br>1 Yes<br>0 No
310	kw_fertility_yn	Section Header: *fertility feeling*<br>Do you feel your fertility is disturbed or limited?	radio, Required<br>1 Yes<br>0 No
311	kw_fertility_adv_yn<br>Show the field ONLY if:<br>[kw_fertility_yn] = '1'	Have you been consulted by your gynecologist regarding this feeling?	radio, Required<br>1 Yes<br>0 No
312	kw_fertility_adv_sc<br>Show the field ONLY if:<br>[kw_fertility_adv_yn] = '1'	Please rate your satisfaction with consultation regarding impaired fertility feeling.<br>*Drag the blue slider and drop it at the desired position.*	slider (number), Required<br>Slider labels: 0, 50, 100<br>Custom alignment: RH
313	kw_fertility_adv_wish<br>Show the field ONLY if:<br>[kw_fertility_adv_yn] = '0'	Would you have wished for a consultation by your gynecologist regarding this?	radio, Required<br>1 Yes<br>0 No
314	kw_satisf_sc	Section Header: *overall satisfaction fertility*<br>Please rate your overall satisfaction with the medical care you receive from your gynecologist regarding fertility?<br>*Drag the blue slider and drop it at the desired position.*	slider (number), Required<br>Slider labels: 0, 50, 100<br>Custom alignment: RH
315	kw_addit_yn	Would you like additional consultation?	radio, Required<br>1 Yes<br>0 No
316	kw_addit_spec<br>Show the field ONLY if:<br>[kw_addit_yn] = '1'	Please select what you would wish to have in addition:	checkbox, Required<br>1 kw_addit_spec___1 More consultation and reassurance<br>2 kw_addit_spec___2 More information providing (such as booklets)<br>3 kw_addit_spec___3 More possibilities to ask questions<br>4 kw_addit_spec___4 More examinations (blood tests, ultrasound, etc.)<br>5 kw_addit_spec___5 More therapy options<br>88 kw_addit_spec___88 Other
317	kw_addit_spec_88<br>Show the field ONLY if:<br>[kw_addit_spec(88)] = '1'	What other additional consultation?<br>*Please specify.*	notes, Required
318	einflussbereich_4_kinderwunsch_complete	Section Header: *Form Status*<br>Complete?	dropdown<br>0 Incomplete<br>1 Unverified<br>2 Complete

319	psy_kb_sorg_yn	Section Header: *body image*<br>Do you worry a lot about the way you look and wish you could think about it less?	radio, Required<br>1 Yes<br>0 No
320	psy_kb_sorg_1h_yn	On a typical day, do you spend more than 1 hour per day worrying about your appearance?	radio, Required<br>1 Yes<br>0 No
321	psy_kb_sorg_prob_yn<br>Show the field ONLY if:<br>[psy_kb_sorg_yn] = '1' or [psy_kb_sorg_1h_yn] = '1'	Are your worries making it hard to do your job or be with family and friends?	radio, Required<br>1 Yes<br>0 No
322	psy_kb_fig	Please look at the following figures and answer the following two questions about them:	descriptive

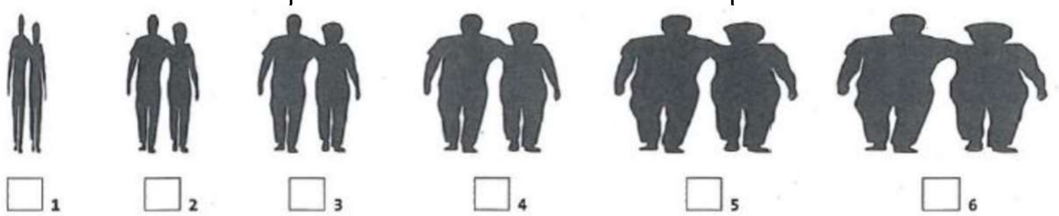

323	psy_kb_fig_ist	Which of the pictures best shows how you see yourself?	dropdown, Required<br>1 1<br>2 2<br>3 3<br>4 4<br>5 5<br>6 6
324	psy_kb_fig_wish	Which of the pictures best shows how you would like to be?	dropdown, Required<br>1 1<br>2 2<br>3 3<br>4 4<br>5 5<br>6 6
325	psy_kb_adv_yn	Have you been consulted by your gynecologist regarding your body image?<br>*Were you asked these or similar questions or was the topic of body image discussed?*	radio, Required<br>1 Yes<br>0 No
326	psy_kb_adv_sc<br>Show the field ONLY if:<br>[psy_kb_adv_yn] ='1'	Please rate your satisfaction with consultation regarding body image.<br>*Drag the blue slider and drop it at the desired position.*	slider (number), Required<br>Slider labels: 0, 50, 100<br>Custom alignment: RH
327	psy_kb_adv_wish<br>Show the field ONLY if:<br>[psy_kb_adv_yn]='0'	Would you have wished for a consultation by your gynecologist regarding body image?	radio, Required<br>1 Yes<br>0 No
328	psy_ess_gew_yn	Section Header: *Eating habits*<br>Does your weight affect the way you feel about yourself?	radio, Required<br>1 Yes<br>0 No
329	psy_ess_satisf_yn	Are you satisfied with your eating patterns?	radio, Required<br>1 Yes<br>0 No
330	psy_ess_adv_yn	Have you been consulted by your gynecologist regarding your eating habits?<br>*Were you asked these or similar questions or was the topic of eating habits discussed?*	radio, Required<br>1 Yes<br>0 No
331	psy_ess_adv_sc<br>Show the field ONLY if:<br>[psy_ess_adv_yn] ='1'	Please rate your satisfaction with consultation regarding eating habits.<br>*Drag the blue slider and drop it at the desired position.*	slider (number), Required<br>Slider labels: 0, 50, 100<br>Custom alignment: RH
332	psy_ess_adv_wish<br>Show the field ONLY if:<br>[psy_ess_adv_yn]='0'	Would you have wished for a consultation by your gynecologist regarding eating habits?	radio, Required<br>1 Yes<br>0 No
333	psy_ad_info	Section Header: *depressive and anxiety symptoms*<br>Over the last 2 weeks, how often have you been bothered by the following problems?	descriptive
334	psy_ad_d1	feeling down, depressed, or hopeless?	dropdown, Required

			5 Constantly (almost every day)<br>4 Often (more than half of days)<br>3 Sometimes (half of days)<br>2 Rarely (some days)<br>1 Never
335	psy_ad_d2	little interest or pleasure in doing things?	dropdown, Required<br>5 Constantly (almost every day)<br>4 Often (more than half of days)<br>3 Sometimes (half of days)<br>2 Rarely (some days)<br>1 Never
336	psy_ad_a1	feeling nervous, anxious or on edge?	dropdown, Required<br>5 Constantly (almost every day)<br>4 Often (more than half of days)<br>3 Sometimes (half of days)<br>2 Rarely (some days)<br>1 Never
337	psy_ad_a2	not being able to stop or control worrying?	dropdown, Required<br>5 Constantly (almost every day)<br>4 Often (more than half of days)<br>3 Sometimes (half of days)<br>2 Rarely (some days)<br>1 Never
338	psy_ad_adv_yn	Have you been consulted by your gynecologist regarding such feelings?	radio, Required<br>1 Yes<br>0 No
339	psy_ad_adv_sc<br>Show the field ONLY if:<br>[psy_ad_adv_yn] = '1'	Please rate your satisfaction with consultation regarding such feelings.<br>*Drag the blue slider and drop it at the desired position.*	slider (number), Required<br>Slider labels: 0, 50, 100<br>Custom alignment: RH
340	psy_ad_adv_wish<br>Show the field ONLY if:<br>[psy_ad_adv_yn] = '0'	Would you have wished for a consultation by your gynecologist regarding such feelings?	radio, Required<br>1 Yes<br>0 No
341	psy_satisf_sc	Section Header: *overall satisfaction mental health and emotional wellbeing*<br>Please rate your overall satisfaction with the medical care you receive from your gynecologist regarding mental health and emotional wellbeing?<br>[illegible]	slider (number), Required<br>Slider labels: 0, 50, 100<br>Custom alignment: RH
342	psy_addit_yn	Would you like additional consultation?	radio, Required<br>1 Yes<br>0 No
343	psy_addit_spec<br>Show the field ONLY if:<br>[psy_addit_yn]='1'	Please select what you would wish to have in addition:	checkbox, Required<br>1 psy_addit_spec___1 More consultation and reassurance<br>2 psy_addit_spec___2 More information providing (such as booklets)<br>3 psy_addit_spec___3 More possibilities to ask questions<br>4 psy_addit_spec___4 More examinations (blood tests, ultrasound, etc.)<br>5 psy_addit_spec___5 More therapy options<br>88 psy_addit_spec___88 Other
344	psy_addit_spec_88<br>Show the field ONLY if:<br>[psy_addit_spec(88)] ='1'	What other additional consultation?<br>*Please specify .*	notes
345	einflussbereich_5_psyche_emotion_complete	Section Header: *Form Status*<br>Complete?	dropdown<br>0 Incomplete<br>1 Unverified<br>2 Complete

346	risk_ass_yn	Has your gynecologist talked to you about risks that may be increased with PCOS?<br>*If you are not sure what is meant by this, you can select "Yes" and look at the subcategories. If there is nothing that applies, you can also switch back to "No".*	radio, Required<br>1 Yes<br>0 No
347	risk_ass_spec<br>Show the field ONLY if: [risk_ass_yn] = '1'	Please select which topics have been addressed:	checkbox, Required<br>1 risk_ass_spec___1 Cardiovascular diseases (e.g. high blood pressure, heart attack, stroke, thrombosis, pulmonary embolism, etc.)<br>2 risk_ass_spec___2 Diabetic disorders (e.g. diabetes, pre-diabetes, gestational diabetes)<br>3 risk_ass_spec___3 Obstructive sleep apnea (pauses in breathing while sleeping)<br>4 risk_ass_spec___4 Endometrial cancer (cancer of the uterine mucosa)<br>88 risk_ass_spec___88 other
348	risk_ass_spec_88<br>Show the field ONLY if: [risk_ass_spec(88)] = '1'	What other risks?<br>*Please specify.*	notes, Required
349	risk_cvrf	Section Header: *Cardiovascular diseases and weight*<br>Do you have any of the following conditions? Please select the applicable ones.	checkbox, Required<br>1 risk_cvrf___1 smoking<br>2 risk_cvrf___2 overweight<br>3 risk_cvrf___3 high cholesterol levels<br>4 risk_cvrf___4 high blood pressure<br>5 risk_cvrf___5 impaired glucose-tolerance or diabetes<br>6 risk_cvrf___6 lack of exercise<br>7 risk_cvrf___7 Cardiovascular diseases in closer family (e.g. heart attack, stroke)<br>0 risk_cvrf___0 None of the above<br>Field Annotation: @NONEOFTHEABOVE=0
350	risk_cvd_yn	Do you have/have you had any cardiovascular disease?<br>*If you are not sure what is meant by this, you can select "Yes" and look at the subcategories. If there is nothing that applies, you can also switch back to "No".*	radio, Required<br>1 Yes<br>0 No
351	risk_cvd_spec<br>Show the field ONLY if: [risk_cvd_yn] = '1'	Please select the applicable ones:	checkbox, Required<br>1 risk_cvd_spec___1 high blood pressure<br>2 risk_cvd_spec___2 heart attack<br>3 risk_cvd_spec___3 stroke<br>4 risk_cvd_spec___4 pulmonary embolism<br>5 risk_cvd_spec___5 thrombosis / embolism (e.g. leg/arm)<br>88 risk_cvd_spec___88 other
352	risk_cvd_spec_88<br>Show the field ONLY if: [risk_cvd_spec(88)] = '1'	What other Cardiovascular diseases?<br>*Please specify.*	notes, Required
353	risk_screen_weight_yn	Is your weight measured regularly by your gynecologist?<br>*Regular means at least once a year.*	radio, Required<br>1 Yes<br>0 No
354	risk_screen_bd_yn	Is your blood pressure measured regularly by your gynecologist?<br>*Regular means at least once a year.*	radio, Required<br>1 Yes<br>0 No
355	risk_screen_chol_yn	Is your blood cholesterol level measured regularly by your gynecologist?<br>*Regular means at least once a year.*	radio, Required<br>1 Yes<br>0 No<br>99 Unknown
356	risk_screen_chol_ha_yn<br>Show the field ONLY if: [risk_screen_chol_yn] = '0' or [risk_screen_chol_yn] = '99'	Is your blood cholesterol level measured regularly by your primary care physician?<br>*Regular means at least once a year.*	radio, Required<br>1 Yes<br>0 No<br>99 Unknown
357	risk_cv_adv_yn	Have you been consulted by your gynecologist regarding increased risk for cardiovascular disease in PCOS and the risk factors for cardiovascular disease?	radio, Required<br>1 Yes<br>0 No

358	risk_cv_adv_sc<br>Show the field ONLY if:<br>[risk_cv_adv_yn] = '1'	Please rate your satisfaction with consultation regarding risk for cardiovascular diseases.<br>*Drag the blue slider and drop it at the desired position.*	slider (number), Required<br>Slider labels: 0, 50, 100<br>Custom alignment: RH
359	risk_cv_adv_wish<br>Show the field ONLY if:<br>[risk_cv_adv_yn] = '0'	Would you have wished for a consultation by your gynecologist regarding risk for cardiovascular diseases?	radio, Required<br>1 Yes<br>0 No
360	risk_screen_bz_yn	Section Header: *glucose tolerance and diabetes*<br>Is your blood glucose level measured regularly by your gynecologist?<br>*Regular here means at least once every three years.*	radio, Required<br>1 Yes<br>0 No<br>99 Unknown
361	risk_screen_bz_ha_yn<br>Show the field ONLY if:<br>[risk_screen_bz_yn] = '0'	Is your blood glucose level measured regularly by your primary care physician or diabetologist?<br>*Regular here means at least once every three years.*	radio, Required<br>1 Yes<br>0 No<br>99 Unknown
362	risk_screen_bz_ogtt_yn<br>Show the field ONLY if: [kw_ss_yn] = '1'	Did you have a oral glucose tolarance test (=oGTT) done before pregnancy??<br>*Fasting blood glucose measurement, then drink glucose solution followed by multiple measurements of glucose*	radio, Required<br>1 Yes<br>0 No<br>99 Unknown
363	risk_screen_bz_ogtt_yn_2<br>Show the field ONLY if: [kw_ss_yn] = '1'	Did you have a oral glucose tolarance test (=oGTT) done during pregnancy??<br>*Fasting blood glucose measurement, then drink glucose solution followed by multiple measurements of glucose*	radio, Required<br>1 Yes<br>0 No<br>99 Unknown
364	risk_bz_adv_yn	Have you been consulted by your gynecologist regarding increased risk for diabetes in PCOS?	radio, Required<br>1 Yes<br>0 No
365	risk_bz_adv_sc<br>Show the field ONLY if:<br>[risk_bz_adv_yn] = '1'	Please rate your satisfaction with consultation regarding risk for diabetes.<br>*Drag the blue slider and drop it at the desired position.*	slider (number), Required<br>Slider labels: 0, 50, 100<br>Custom alignment: RH
366	risk_bz_adv_wish<br>Show the field ONLY if:<br>[risk_bz_adv_yn] = '0'	Would you have wished for a consultation by your gynecologist regarding risk for diabetes?	radio, Required<br>1 Yes<br>0 No
367	risk_osas_symp	Section Header: *Obstructive sleep apnea*<br>Please select the applicable ones:	checkbox, Required<br>1 risk_osas_symp___1 snoring<br>2 risk_osas_symp___2 Breathing pauses during sleep (e.g. observed by partner)<br>3 risk_osas_symp___3 waking unrefreshed from sleep<br>4 risk_osas_symp___4 daytime sleepiness<br>5 risk_osas_symp___5 morning headaches<br>6 risk_osas_symp___6 Concentration difficulties<br>0 risk_osas_symp___0 None of the above<br>Field Annotation: @NONEOFTHEABOVE=0
368	risk_osas_yn	Are you known to have sleep apnea syndrome?<br>*Select "Yes" only if this has been diagnosed by a physician.*	radio, Required<br>1 Yes<br>0 No
369	risk_osas_psg_yn	Have you ever been to a sleep medicine specialist or sleep lab?	radio, Required<br>1 Yes<br>0 No
370	risk_osas_adv_yn	Have you been counseled by your gynecologist regarding increased risk for sleep apnea (breathing pauses during sleep)?	radio, Required<br>1 Yes<br>0 No
371	risk_osas_adv_sc<br>Show the field ONLY if:<br>[risk_osas_adv_yn] = '1'	Please rate your satisfaction with consultation regarding risk for sleep apnea.<br>*Drag the blue slider and drop it at the desired position.*	slider (number), Required<br>Slider labels: 0, 50, 100<br>Custom alignment: RH
372	risk_osas_adv_wish<br>Show the field ONLY if:<br>[risk_osas_adv_yn] = '0'	Would you have wished for a consultation by your gynecologist regarding risk for sleep apnea?	radio, Required<br>1 Yes<br>0 No
373	risk_endca_yn	Section Header: *Endometrial Cancer*<br>Do you suffer or have you suffered from endometrial cancer?<br>*Endometrial cancer = cancer of the mucus ("endometrium") of the uterus*	radio, Required<br>1 Yes<br>0 No

374	risk_endca_adv_yn	Have you been advised by your gynecologist regarding slightly increased risk for endometrial cancer?	radio, Required<br>1 Yes<br>0 No
375	risk_endca_adv_sc<br>Show the field ONLY if:<br>[risk_endca_adv_yn] = '1'	Please rate your satisfaction with consultation regarding slightly increased risk for endometrial cancer.<br>*Drag the blue slider and drop it at the desired position.*	slider (number), Required<br>Slider labels: 0, 50, 100<br>Custom alignment: RH
376	risk_endca_adv_wish<br>Show the field ONLY if:<br>[risk_endca_adv_yn] = '0'	Would you have wished for a consultation by your gynecologist regarding risk for slightly increased risk for endometrial cancer?	radio, Required<br>1 Yes<br>0 No
377	risk_satisf_sc	Section Header: *overall satisfaction prevention and monitoring*<br>Please rate your overall satisfaction with the medical care you receive from your gynecologist regarding prevention and monitoring?<br>*Drag the blue slider and drop it at the desired position.*	slider (number), Required<br>Slider labels: 0, 50, 100<br>Custom alignment: RH
378	risk_addit_yn	Would you like additional consultation?	radio, Required<br>1 Yes<br>0 No
379	risk_addit_spec<br>Show the field ONLY if:<br>[risk_addit_yn] = '1'	Please select what you would wish to have in addition:	checkbox, Required<br>1 risk_addit_spec___1 More consultation and reassurance<br>2 risk_addit_spec___2 More information providing (such as booklets)<br>3 risk_addit_spec___3 More possibilities to ask questions<br>4 risk_addit_spec___4 More examinations (blood tests, ultrasound, etc.)<br>5 risk_addit_spec___5 More therapy options<br>88 risk_addit_spec___88 Other
380	risk_addit_spec_88<br>Show the field ONLY if:<br>[risk_addit_spec(88)] = '1'	What other additional consultation?<br>*Please specify.*	notes, Required
381	risikomonitoring_complete	Section Header: *Form Status*<br>Complete?	dropdown<br>0 Incomplete<br>1 Unverified<br>2 Complete

Instrument: **final questions** (schlussfragen) Enabled as survey

382	sf_satisf_sc	If you now consider all the areas in which you are affected by your PCOS as a whole:<br>Please rate your satisfaction in general with the overall advice regarding PCOS from your gynecologist?<br>*Drag the blue slider and drop it at the desired position.*	slider (number), Required<br>Slider labels: 0, 50, 100<br>Custom alignment: RH
383	sf_addit_yn	Would you like additional consultation overall?	radio, Required<br>1 Yes<br>0 No
384	sf_addit_spec<br>Show the field ONLY if:<br>[sf_addit_yn] = '1'	Please select what you would wish to have in addition:	checkbox, Required<br>1 sf_addit_spec___1 More consultation and reassurance<br>2 sf_addit_spec___2 More information providing (such as booklets)<br>3 sf_addit_spec___3 More possibilities to ask questions<br>4 sf_addit_spec___4 More examinations (blood tests, ultrasound, etc.)<br>5 sf_addit_spec___5 More therapy options<br>88 sf_addit_spec___88 Other
385	sf_addit_spec_88<br>Show the field ONLY if:<br>[sf_addit_spec(88)] = '1'	What other additional consultation?<br>*Please specify.*	notes, Required
386	sf_satisf_change_yn	Did this survey change anything about your satisfaction with your gynecologist?	radio, Required<br>1 Yes<br>0 No
387	sf_satisf_change_before<br>Show the field ONLY if:<br>[sf_satisf_change_yn] = '1'	Please rate your satisfaction before the survey.<br>*Drag the blue slider and drop it at the desired position.*	slider (number), Required<br>Slider labels: 0, 50, 100<br>Custom alignment: RH
388	sf_satisf_change_spec<br>Show the field ONLY if:<br>[sf_satisf_change_yn] = '1'	To what extent has the survey changed your opinion?<br>*Please specify.*	notes, Required<br>Custom alignment: RH
389	sf_satisf_change_info<br>Show the field ONLY if:<br>[sf_satisf_change_yn] = '1'	We would like to point out that this survey covers all possible points of care according to the new guidelines. If your gynecologist does not do everything the same way, this does not mean that he/she does it badly.<br>If you have any uncertainties or feel you are receiving bad care after this survey, please contact your gynecologist and discuss the situation with her/him. There is certainly a solution for this.	descriptive
390	sf_zus_yn	Was there a question missing from this survey that you felt was important?	radio, Required<br>1 Yes<br>0 No
391	sf_zus_spec<br>Show the field ONLY if:<br>[sf_zus_yn] = '1'	Please write down the missing question(s):	notes, Required<br>Custom alignment: RH
392	sf_note_yn	Is there anything else you would like to say?	radio, Required<br>1 Yes<br>0 No
393	sf_note_spec<br>Show the field ONLY if:<br>[sf_note_yn] = '1'	Comment:	notes, Required<br>Custom alignment: RH
394	sf_recr_spec	How did you come across this survey?	checkbox, Required<br>1 sf_recr_spec___1 resident gynecologist<br>2 sf_recr_spec___2 hospital gynecologist<br>3 sf_recr_spec___3 Poster / Flyer<br>4 sf_recr_spec___4 Social Media<br>5 sf_recr_spec___5 University newsletter<br>6 sf_recr_spec___6 Internet forum<br>7 sf_recr_spec___7 Selfhelp group<br>8 sf_recr_spec___8 Friends / Acquaintance<br>88 sf_recr_spec___88 other

395	sf_recr_spec_88<br>Show the field ONLY if:<br>[sf_recr_spec(88)] = '1'	What other possibility?<br>*Please specify.*	notes, Required
396	sf_recr_spec4<br>Show the field ONLY if:<br>[sf_recr_spec(4)] = '1'	Through which social media channel?	checkbox, Required<br>1 sf_recr_spec4___1 Facebook<br>2 sf_recr_spec4___2 Instagram<br>3 sf_recr_spec4___3 LinkedIn<br>88 sf_recr_spec4___88 other
397	sf_recr_spec4_88<br>Show the field ONLY if:<br>[sf_recr_spec4(88)] = '1'	What other social media channel?<br>*Please specify.*	notes, Required
398	schlussfragen_complete	Section Header: *Form Status*<br>Complete?	dropdown<br>0 Incomplete<br>1 Unverified<br>2 Complete
