# Supplementary material for: Unmet clinical needs in women with polycystic ovary syndrome in regard to mental health: a cross-sectional study
Source: Arch Gynecol Obstet. 2024 Mar 11;309(5):2115–26. doi: 10.1007/s00404-024-07452-y (PMC11018694; doi:10.1007/s00404-024-07452-y)
Supplement: Supplementary file 4 — Supplementary file4 (PDF 626 KB) [file 404_2024_7452_MOESM4_ESM.pdf]

Archives of Women's Mental Health

Unmet clinical needs in women with polycystic ovary syndrome in regard to mental health – a cross-sectional study

Sourouni Marina, M. D, Estermann Julia, M. D., Bitterlich Norman, Ph. D, Weidlinger Susanna, M. D., Bachmann Annette, M. D., Stute Petra, M.D.

Corresponding author:

Professor Dr. med. Petra Stute, M. D.

Department of Obstetrics and Gynaecology

University Hospital Inselspital

Friedbuehlstrasse 19, 3010 Bern, Switzerland

E-mail: petra.stute@insel.ch

Telephone: (00)41-31-632-1303

Fax: (00)41-31-632-1305

ORCID:0000-0002-5591-1552

PCOS

Polyzystisches Ovarialsyndrom

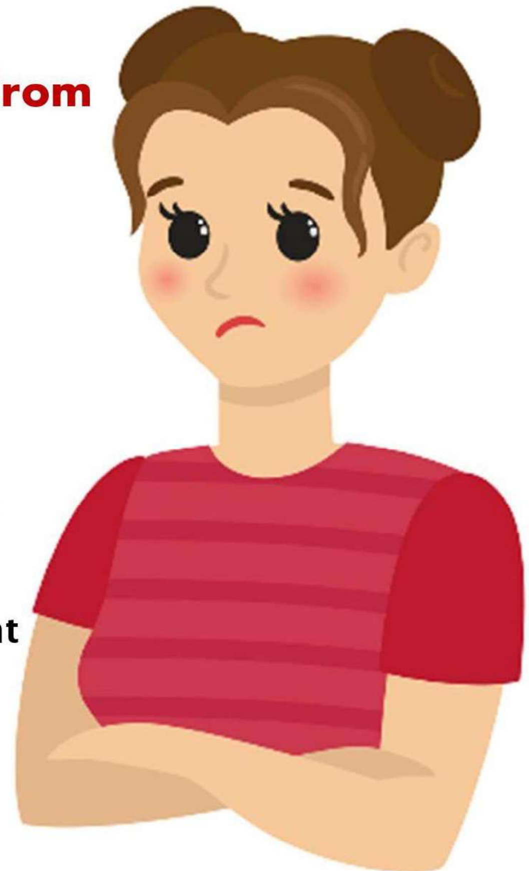

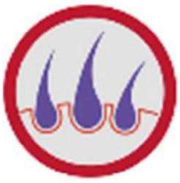

übermässige Körperbehaarung

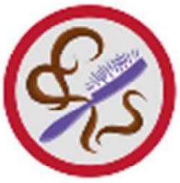

Haarausfall

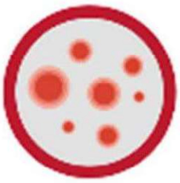

Akne

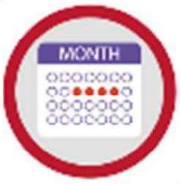

unregelmässige Menstruation

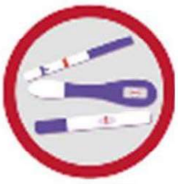

unerfüllter Kinderwunsch

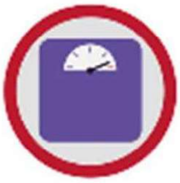

Übergewicht

Treffen diese Symptome auf Sie zu oder ist bei Ihnen PCOS bekannt?

Dann sind Sie hier genau richtig!

STUDIEN-TEILNEHMERINNEN FÜR ONLINE-UMFRAGE GESUCHT

Wir möchten wissen, wie gut Frauen mit PCOS von Ihren Frauenärzten/innen betreut werden. Dazu ist IHRE Meinung gefragt! Auch Frauen die sich von den Symptomen angesprochen fühlen, aber noch keine Diagnose erhalten haben, werden gesucht!

Sie leisten einen wichtigen Beitrag zur Verbesserung der Betreuung und haben die Möglichkeit, eine Auswertung der Daten zu erhalten.

Die Umfrage dauert ca. 15-20 Minuten. Alle Daten werden anonym erfasst.

https://is.gd/pcos_study
